# Mental health of internally displaced persons: a meta-analysis

**DOI:** 10.1136/bmjgh-2025-023173

**Published:** 2026-06-04

**Authors:** Ahlke Kip, Angela Nickerson, Philippa Specker, Anna Langiu, Thao My Thi Nguyen, Dana Churbaji, Nexhmedin Morina

**Affiliations:** 1 Medical Psychology and Medical Sociology, University of Freiburg, Freiburg, Germany; 2 School of Psychology, University of New South Wales, Sydney, New South Wales, Australia; 3 Institute of Psychology, University of Münster, Münster, Germany

**Keywords:** Traumatology, Mental Health & Psychiatry, Public Health, Systematic review

## Abstract

**Introduction:**

Although most forcibly displaced individuals remain within their country of origin, internally displaced persons (IDPs) remain under-represented in mental health research. We estimated the prevalence of mental disorders among IDPs and compared prevalence and psychosocial functioning with non-displaced controls.

**Methods:**

We searched MEDLINE, PsycINFO and Web of Science for peer-reviewed reports published until 20 June 2025 in any language. We included studies on point prevalence of mental disorders in adult IDPs as assessed with validated measurements based on the International Classification of Diseases or Diagnostic and Statistical Manual of Mental Disorders. Studies had to assess mental disorders in at least 100 participants at least 1 month postdisplacement. For studies that included non-displaced control groups, we also assessed other mental-health-related outcomes. All analyses used a random effects model. The protocol was registered with PROSPERO, CRD42024596202.

**Results:**

We identified 20774 unique study reports, of which 132 (124 independent datasets and 195 741 participants, with 61.3% female IDPs) met our inclusion criteria. The estimated pooled prevalence of current post-traumatic stress disorder (PTSD) was 39.8% (95% CI 34.9% to 44.8%; I²=98.9%), depression 35.8% (95% CI 27.0% to 45.8%; I²=99.3%), generalised anxiety disorder 29.4% (95% CI 20.3% to 40.5%; I²=99.4%), insomnia 38.6% (95% CI 33.2% to 44.2%; I²=95.5%), panic disorder 6.0% (95% CI 2.7% to 12.9%; I²=95.9%), alcohol use disorder 2.2% (95% CI 0.1% to 6.2%; I²=97.3%) and suicidality 9.2% (95% CI 1.3% to 44.1%; I²=99.2%). The odds of PTSD and depression compared with non-displaced controls were increased by 70%–117% and IDPs reported significantly higher somatic complaints and general distress and lower psychosocial well-being. Displacement cause and socioeconomic conditions in the affected countries insufficiently explained the large heterogeneity in findings. High risk of selection and outcome measurement bias was observed.

**Conclusions:**

The results highlight the mental health burden in IDPs, which calls for scalable and sustainable interventions. The findings further indicate that estimates vary substantially across contexts.

**WHAT IS ALREADY KNOWN ON THIS TOPIC**

- Previous meta-analyses have reported high prevalence of mental disorders in overall displaced populations. However, comparatively little attention has been directed towards internally displaced persons (IDPs), even though most forcibly displaced individuals remain within their country of origin.

**WHAT THIS STUDY ADDS**

- This is the first comprehensive meta-analysis on the mental health of IDPs to include data on all mental disorders and global regions, encompassing all displacement causes and comparing outcomes of IDPs to non-displaced controls.
- Our findings suggest that beyond post-traumatic stress disorder and depression, generalised anxiety, insomnia and suicidality are likewise highly prevalent among IDPs. IDPs not only report a higher prevalence of mental disorders but also higher general distress and lower psychosocial well-being such as quality of life and social functioning compared with non-displaced controls with similar backgrounds.

**HOW THIS STUDY MIGHT AFFECT RESEARCH, PRACTICE OR POLICY**

- The increased odds of mental disorders in IDPs compared with non-displaced controls underscore the association of displacement status and mental health across highly heterogeneous contexts.
- The findings highlight the need to increase IDPs’ access to mental healthcare services, particularly in low-income countries. Sleep disturbances may provide an early entry point for systematic mental health screenings, which should also encompass the assessment of suicide risk.

## Introduction

 Internally displaced persons (IDPs), resettled within national borders, represent the largest share of forcibly displaced populations worldwide with recent estimates of 73.5 million people.^1^ Their number has doubled since 2018, reaching a new peak in 2024, and is expected to rise further due to ongoing conflicts and climate-related pressures.^2 3^ Internal displacement is therefore one of the most pressing societal and public health issues of our time.

Forced displacement may be triggered by specific events of limited duration like tropical storms or occur in protracted contexts such as armed conflicts. It is often marked by ongoing exposure to adversity, disrupted social networks and insecurity.^4^ Displacement has been identified as a significant predictor of mental ill-health following disaster exposure.^5^ Extensive meta-analytic evidence has accumulated on the prevalence of mental disorders—particularly post-traumatic stress disorder (PTSD) and depression—among displaced populations, with estimates of 30%–50% for PTSD^6 7^ and 26%–30% for depression.^7 8^ However, previous work mainly targeted a certain global region (most often Africa^6 9^), focused on displacement following man-made disasters such as armed conflicts,^10^ did not compare outcomes between displaced persons and non-displaced controls, and did not differentiate between international refugees, asylum-seekers and IDPs^7 8^ or focused solely on refugees.^11^ While refugees and IDPs share certain experiences, they also differ in the nature of cumulative stressors. Remaining in their country of origin, IDPs lack international protection and humanitarian assistance, often resulting in protracted accommodation in temporary resettlements with restricted access to education and healthcare.^10 12^ Substantial variation in rates of mental disorders has been observed across studies on IDPs,^13^ possibly due to different displacement causes and varying socioeconomic conditions of affected countries, which influence cumulative stressors, mental healthcare, stigmatisation of mental illhealth, and/or expectations related to institutional responsiveness.

We aimed to provide estimates of the prevalence of mental disorders in IDPs. We considered the displacement cause and the income status of countries affected by displacement to account for contextual differences in displacement. To increase our understanding of the impact of displacement, we furthermore compared prevalence of mental disorders and psychosocial well-being of displaced individuals with non-displaced controls exposed to similar events.

## Methods

The aims and methods of this meta-analysis were registered with the PROSPERO database (CRD42024596202) and the reporting follows the Preferred Reporting Items for Systematic Reviews and Meta-Analyses (PRISMA^14^). Patients or members of the public were not involved in the development of the research question or the conduct of the meta-analysis.

### Search strategy and selection criteria

A systematic database search was conducted in MEDLINE, PsycINFO and Web of Science to identify all publications up to 20 June 2025. The search was restricted to peer-reviewed journal articles with no restrictions regarding language, publication date, or geographical location. Search terms indicative of mental health (eg, “depress*”, “bereave*”) were searched in title, abstract, and keywords of articles. Search terms indicative of displacement (eg, “relocat*”, “temporary ho*”) were searched across all fields to capture studies with subgroup analyses based on displacement status (online supplemental 1). Title and abstract screening was conducted using Rayyan,^15^ where duplicates were removed. Three independent reviewers rated 140 pilot studies to assess inter-rater agreement, with an almost perfect result (99.5%). The remaining hits were divided between the reviewers with unclear cases being discussed. We furthermore screened the reference lists of 23 systematic reviews and meta-analyses on related topics to supplement database searches (online supplemental 2).

Studies with an internally displaced sample only were included if they (1) reported point prevalence estimates, (2a) of mental disorders according to the International Classification of Diseases (ICD; any version^16^) or the Diagnostic and Statistical Manual of Mental Disorders (any version^17^) or (2b) suicidality (3) based on a validated clinician-administered interview or self-report scale. Moreover, the internally displaced sample had to (4) have been displaced for at least one month, (5) have a mean age of at least 18 years and (6) consist of at least 100 participants. Studies that compared IDPs with non-displaced controls were moreover included if they reported mean scores of outcomes indicative of psychosocial well-being and general distress or odds ratios (OR) for probable mental disorders. The minimum number of participants was applied to both sample groups. Studies that reported combined data for refugees, asylum seekers, and IDPs were excluded, unless authors provided separated data. We further excluded studies with samples that (1) were evacuated before being exposed to a potentially traumatic event such as a natural hazard or armed conflict, (2) had returned to their property at the time the study was conducted or rebuilt their homes, (3) were displaced based on governmental planned relocation such as due to gentrification or from illegal settlements in occupied territories and (4) were displaced due to individual traumatic events such as intimate partner violence. These criteria were applied to ensure a more homogeneous definition of current displacement, as these populations may differ substantially in terms of living conditions, ongoing stressors, or access to resources. To reduce the risk of inflated prevalence estimates, we furthermore excluded samples that were recruited based on the presence of certain symptoms (eg, nightmares), in psychosocial support programmes, primary care settings, humanitarian services or in hospitals. Studies were moreover excluded if they focused on individuals without pre-existing mental disorder (except those interfering with study participation such as dementia), thus reporting on incidences rather than prevalences.

Data from primary studies were extracted by a set of two reviewers with data checks being conducted by an additional set of two reviewers. Extracted data included information on the study (eg, setting, country), displacement reason (eg, type, stage), sample (eg, percentage female participants, age, education), and outcome (eg, measurement, prevalence estimates, mean symptom severity). The socioeconomic condition of affected countries was coded based on the World Bank’s income status.^18^ The latest assessment was extracted in longitudinal studies given the higher clinical relevance, provided a maximum drop-out rate of 30%. The percentage of participants with low education level was coded with a cut-off of 8 years of education, excluding religious schools. We contacted authors from 46 studies twice to request any missing information necessary for analysis (eg, raw data in case only OR were reported); 27 responded and 12 provided additional unpublished data.

### Data analysis

All analyses were conducted in RStudio (V.2025.9.0.387^19^) with a minimum of four independent data points.^20^ Outcomes included pooled prevalence, OR for the comparison of prevalence between IDPs and non-displaced controls, and Hedges’ *g* for the comparison of additional health-related outcomes. Prevalence estimates were logit-transformed prior to meta-analysis with pooled estimates back-transformed for interpretation. To account for heterogeneity between studies regarding sample, displacement reason, or outcome measurement, random-effects models were applied across all analyses. Heterogeneity was assessed using Cochran’s Q statistic and Higgins’ I². Additionally, 95% prediction intervals (PI) were calculated for all prevalence estimates that reflect the expected range of effects in future settings, accounting for between-study variability. To investigate possible sources of heterogeneity, we examined moderation effects of displacement reason (armed conflict vs natural hazard) and income status of affected country (high, upper middle, lower middle, low). Publication bias was assessed using Egger’s test^21^ for funnel plot asymmetry for analyses including at least 10 studies.^22^ Robustness of results was tested by means of leave-one-out- and outlier-adjusted analyses, and sensitivity analyses were calculated to investigate the influence of risk of bias on results. For analyses including dependent data (eg, studies that reported on physical-related and mental-health-related quality of life), we applied three-level models.

### Risk of bias

Four key domains were considered to assess the methodological quality of included studies based on previous meta-analyses.^23^ Risk of bias arising from participant selection was examined by means of participation rate (<70%), critical exclusion criteria (eg, illiterate individuals), recruitment strategy (ie, non-probability sampling), and representativeness of the sample (eg, focus on pregnant women). Performance bias included the type of outcome measurement, the validity of translations, and the training of assessors. Attrition bias was evaluated by examining information on the extent and handling of missing outcome data (≥5%). Finally, reporting bias considered the availability of a preregistered protocol and inconsistencies between registered and reported outcomes. The risk of bias was rated as high, low or unclear, with an additional moderate risk of bias category for participant selection.

## Results

### Study characteristics

**Figure 1:**
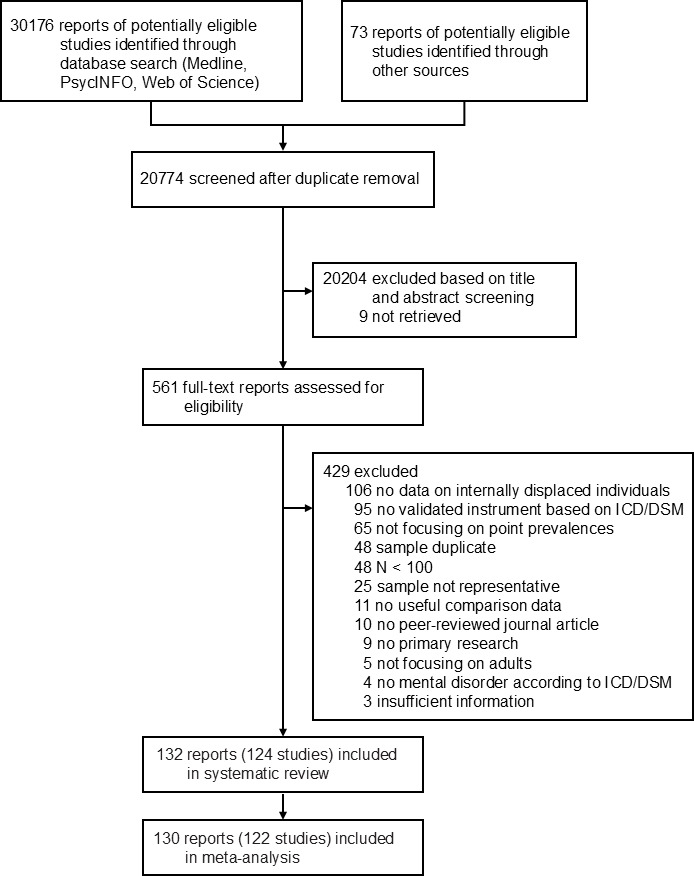
Study selection. A list of excluded studies based on full-text review with reasons for exclusion is provided in online supplemental 4. DSM, Diagnostic and Statistical Manual of Mental Disorders; ICD, International Classification of Diseases.

**Figure 2:**
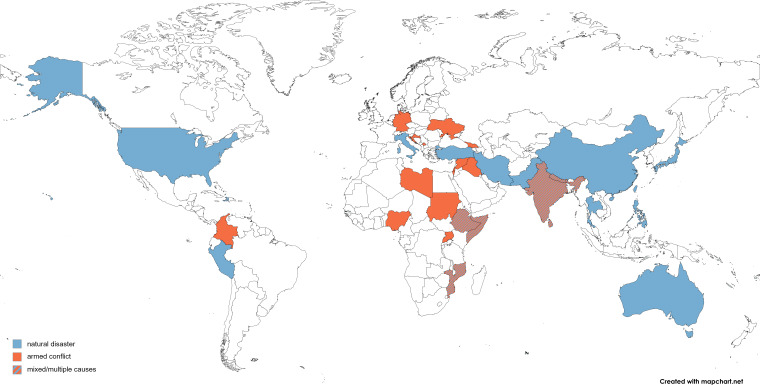
World map of represented countries.

A total of 20 774 studies identified from database searches and other sources were screened for eligibility, resulting in the inclusion of 124 studies from 132 reports (see figure 1) with 195 741 unique participants. Study characteristics are presented in online supplemental 3 with corresponding references of included studies in online supplemental 4. A list of excluded studies with reasons for exclusion is displayed in online supplemental 5. The income status distribution showed a balanced representation, with 31 studies (25.0%) conducted in low, 40 studies (32.3%) conducted in lower-middle, 21 studies (16.9%) in upper-middle and 25 studies (20.2%) conducted in high-income countries (for 5.6% of studies, income status could not be determined because the study time was not reported or there was a change in status throughout the study; see figure 2). Study participants were mainly displaced due to armed conflicts (54.8%), with 75.4% of conflicts still ongoing when the study was conducted, or natural disasters (39.7%). 35 (27.8%) studies included a non-displaced control group. Control samples had a mean age of 46.5 years, with 64.9% identifying as female. Displaced samples had a mean age of 39.6 years, with 61.3% identifying as female (only one study reported on non-binary gender). Almost half (44.8%) of the displaced participants belonged to a religious or ethnic minority in their country and nearly half (48.9%) had primary education or less. Study setting was not reported or mixed in 51.6% of studies (including online or postal surveys), whereas 27.4% were conducted in IDP camps and 21.0% in “resettlement sites” (of which 3.2% permanent, 15.3% temporary, 2.4% not reported). According to the study reports, most IDPs were accommodated in temporary housing facilities (28.1%) and tents (19.3%), other reported accommodations included containers, trailers, or informal shelters. The most frequently reported adverse experiences among IDPs were the destruction of one’s house, the loss of loved ones, a lack of food, water or medical care, direct exposure to combat or violence, and witnessing death or injury.

### Prevalence of mental disorders among IDPs

The prevalence of current PTSD across 98 studies and 58 636 IDPs was 39.8% (95% CI 34.9% to 44.8%), with considerable heterogeneity between studies (I²=98.9%; 95% PI 7.7% to 84.0%). Across studies reporting prevalence by gender, the prevalence among female IDPs was significantly higher compared with male IDPs (47.7% vs 37.4%; OR=1.42, 95% CI 1.11 to 1.82; k=30, online supplemental 6). Prevalences were moreover significantly higher in samples displaced by armed conflict (46.3%) compared with natural hazards (31.8%; p=0.003), but heterogeneity between studies remained high after considering this moderator. No differences were observed for the socioeconomic condition of affected countries.

The prevalence of current depression across 37 studies and 24 085 IDPs was 35.8% (95% CI 27.0% to 45.8%), with considerable study heterogeneity (I²=99.3%, 95% PI 3.9% to 88.6%). Only four studies reported prevalences for male IDPs separately with an aggregated prevalence of 67.3%, whereas the prevalence for female IDPs was reported in six studies with an aggregated estimate of 66.1%. The gender difference was not significant (OR=1.45; 95% CI 0.67 to 3.13). No significant difference was observed between IDPs displaced by armed conflict vs natural hazards (p=0.342), but the prevalence was significantly higher among IDPs in low-income countries (53.4%) compared with lower-middle- (27.0%) and high-income countries (24.6%). Yet, residual heterogeneity between studies remained considerable.

The prevalence of current generalised anxiety disorder across 22 studies and 16 718 IDPs was 29.4% (95% CI 20.3% to 40.5%), with considerable heterogeneity between studies (I²=99.4%, 95% PI 3.3% to 83.5%). Four studies reported prevalence estimates for female IDPs with an aggregated prevalence of 39.1%. Prevalence estimates among male IDPs were only reported in three studies with all primary studies reporting estimates of below 33%, yet no statistical comparison was calculated. Despite larger estimates of generalised anxiety among IDPs in the context of armed conflicts (32.4% vs 13.1%), the difference remained non-significant (p=0.088), similar to the comparison of different socioeconomic conditions in the affected countries (p=0.851).

Prevalence estimates for other mental disorders were limited, with only a few being assessed in at least four studies: Preliminary estimates were 38.6% for current insomnia (95% CI 33.2% to 44.2%; 95% PI 22.3% to 57.9%; k=6; n=14 017), 6.0% for panic disorder (95% CI 2.7% to 12.9%; 95% PI 0.3% to 55.2%; k=4; n=3071) and 2.2% for alcohol use disorder (95% CI 0.1% to 6.2%; 95% PI 0.1% to 51.6%; k=4; n=5885). Moreover, across four studies with 2871 IDPs, the prevalence of suicidality (including ideation, plans and attempts as assessed with a clinical interview) was estimated at 9.2% (95% CI 1.3% to 44.1%, 95% PI 0.0% to 99.4%). Additional mental disorders not meta-analytically examined included prolonged grief disorder (k=3; prevalence 29%–76%), complex PTSD (k=3; prevalence 8%–33%), substance use disorder (k=3; prevalence 1%–13%), dysthymia (k=3; prevalence 2%–20%), agoraphobia (k=3; prevalence 4%–39%), social phobia (k=2; prevalence 2%–14%), psychotic disorders (k=2; prevalence 0%–1%), obsessive -compulsive disorder (k=2; prevalence 2%–5%) and bipolar disorder or manic/hypomanic episodes (k=3; prevalence 0%–3%; online supplemental 7).

### Publication bias and sensitivity analyses

No bias arising from small study effects could be observed across PTSD (*t*=0.19; p=0.852), depression (*t*=−0.54; p=0.592) and generalised anxiety disorder (*t*=−0.85; p=0.404), although these results should be interpreted cautiously in light of the substantial between-study heterogeneity.^24^ After excluding statistical outliers from analyses, prevalences remained largely unchanged (PTSD 40.3%, depression 33.9, generalised anxiety disorder 32.3%), and although heterogeneity decreased, it remained substantial. Omitting individual studies did not change results notably (PTSD 39.0%–40.6%, depression 33.7%–37.9%, generalised anxiety disorder 27.2%–31.9%). Analyses were moreover repeated omitting studies with samples displaced by World War II due to the distinct character and study period of those studies, with results remaining largely unchanged (PTSD 40.4%, depression 38.2%). Details on sensitivity analyses can be found in online supplemental 8.

### Mental health among IDPs compared with non-displaced controls

**Figure 3:**
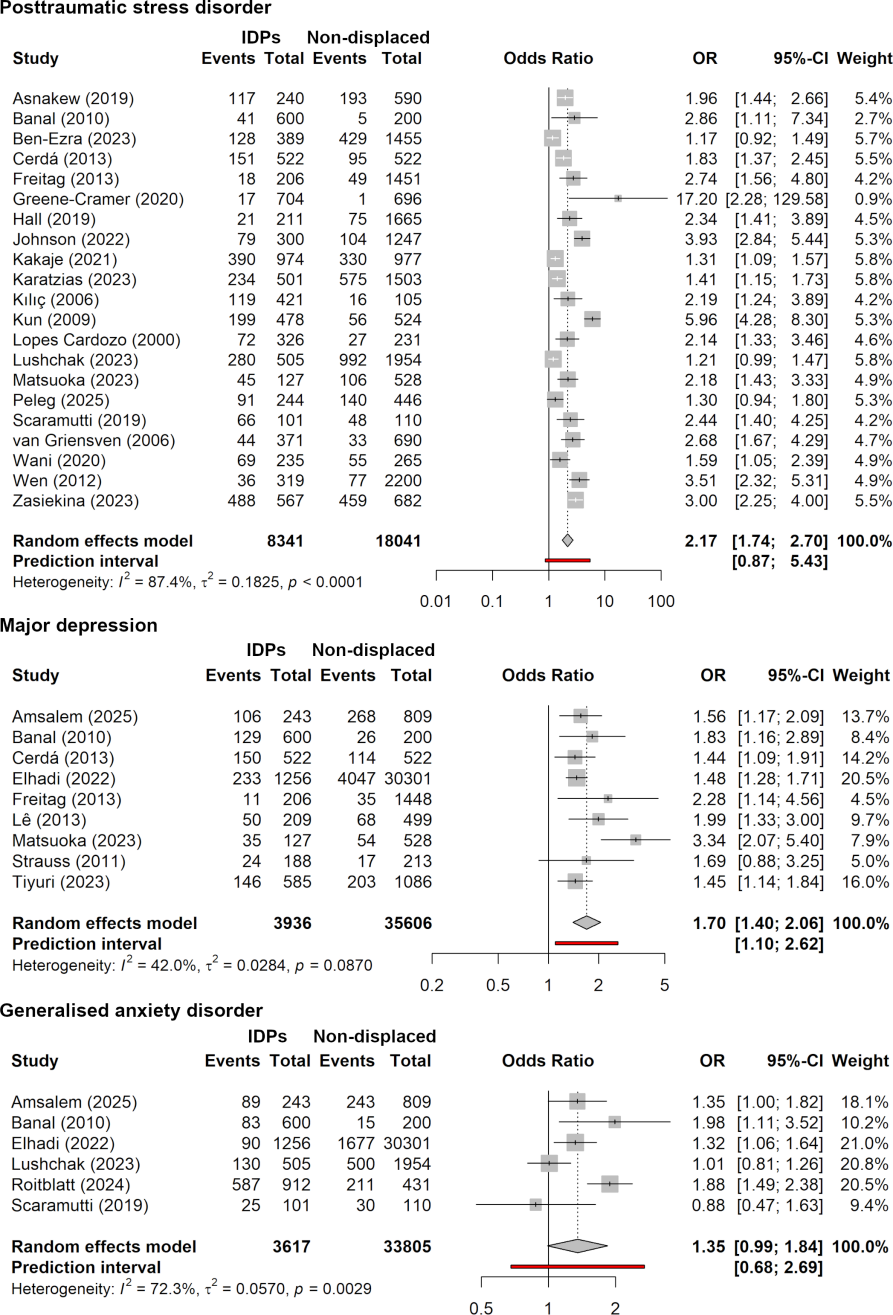
Forest plot of prevalences in IDPs compared with non-displaced controls. IDPs, internally displaced persons; PTSD, post-traumatic stress disorder.

When comparing IDPs with non-displaced individuals, the odds of PTSD were more than twice as high in the IDP samples (OR=2.17; 95% CI 1.74 to 2.70) across 21 studies with 8341 IDPs and 18 041 controls (figure 3). The difference in symptom severity was also significant and moderate to large in size (*g*=0.65; 95% CI 0.02 to 1.27). The odds of depression were significantly increased by 70% among the IDPs across nine studies with 3936 IDPs and 35 606 controls (OR=1.70; 95% CI 1.40 to 2.06) with significantly more severe symptoms observed in IDPs with a small effect (*g*=0.26; 95% CI 0.06 to 0.47). The difference for generalised anxiety disorder narrowly missed statistical significance (OR=1.35; 95% CI 0.99 to 1.84; k=6; n=3617 IDPs and 33 805 controls; p=0.055) and similarly, no significant difference in symptom severity was observed (g=0.56; 95% CI −0.24 to 1.36). These aggregated crude OR appear similar to the adjusted OR reported in primary studies (online supplemental 9), except depression, for which the adjusted primary results were non-significant. Finally, compared with non-displaced controls, IDPs reported significantly higher nonspecific symptoms (including somatic complaints and general distress; *g*=0.33; 95% CI 0.07 to 0.59) and significantly lower psychosocial well-being (including quality of life, life satisfaction, social functioning or posttraumatic growth; *g*=−0.17; 95% CI −0.27 to −0.08). Most studies explicitly indicated that the non-displaced sample had been exposed to the same event as the displaced group (see online supplemental 10 for details). Yet, sociodemographic data were mainly reported for the overall sample and were not disaggregated by displacement status (see online supplemental 3).

### Risk of bias assessment

In terms of methodological quality, 60.5% of studies had a high risk of bias regarding selection of participants while 16.1% had a low risk of bias. Pooled prevalence estimates differed by risk of selection bias (PTSD 43.6% high, 37.7% moderate, 35.9% low risk; depression 39.4% high, 36.3% low risk; generalised anxiety disorder 27.5% high risk, 48.9% low risk), although the differences were not statistically significant (*p*s 0.120–0.779). Moreover, the vast majority of studies had a high risk of bias regarding outcome measurement (91.9%). Restricting analyses to studies of low risk of bias (ie, use of (semi)structured interviews, adequate training or supervision of interviewers, and confirmed validity and reliability of the language versions used) yielded a PTSD prevalence of 28.6% (*k*=4 studies). For the remaining mental disorders and comparisons, an insufficient number of studies were rated as low risk of bias. The influence of the measurement tool on results was furthermore assessed by separating analyses into self-report versus clinician-administered instruments. Results indicate lower estimates for diagnostic interviews (PTSD 40.1% vs 32.3%, depression 32.8% vs 20.7%, generalised anxiety disorder 29.7% vs 17.2%), although differences were not statistically significant (*p*s 0.120-.0779). Risk of bias arising from incomplete outcome data or selective outcome reporting remained unclear in most cases (82.3% and 98.4%) as studies did not report on their handling of missing outcome data and did not cite a publicly available study protocol.

## Discussion

This is the first meta-analysis to comprehensively assess mental disorders in IDPs and compare the mental health and psychosocial well-being of IDPs to non-displaced individuals from the same region. Pooled prevalence estimates suggest a substantial burden of PTSD, depression, generalised anxiety disorder, insomnia and suicidality among IDPs, with considerable variation across individual studies and populations. Across all studies, the likelihood of PTSD and depression was 70%–117% greater than in non-displaced controls, while psychosocial well-being was significantly lower.

According to the latest WHO estimates, 22% of conflict-affected populations—including those remaining in their country of origin or residing in neighbouring countries—suffer from a mental disorder.^25^ However, a recent meta-analysis reported depression and PTSD prevalence estimates of 23% and 26%, respectively.^26^ Both numbers are substantially increased compared with estimates in the general population,^27^ but lower than our pooled prevalence among IDPs. IDPs and non-displaced conflict-affected populations share the experience of potentially traumatic events such as the loss of loved ones or witnessing violence, a disruption of local infrastructure or economic hardship. Displacement involves the abrupt loss of home and property, prolonged housing instability, disruption of social networks and uncertainty about return or resettlement.^4^ Importantly, the considerable between-study heterogeneity and wide PIs indicate that the prevalence of mental disorders in IDPs varies markedly across settings, including different global regions, durations of displacement or types of conflicts. Compared with refugees, IDPs often face administrative invisibility and a lack of formal cross-border international protection,^12^ while their greater proximity to ongoing conflict or danger may further undermine their sense of safety and security. The high proportion of IDPs suffering from mental disorders is particularly concerning given the large global population of IDPs^1^ and high comorbidity rates, which were estimated at 55% in conflict-affected populations.^26^ We observed the highest pooled prevalence for insomnia, which was more than twice as high as estimates from the general population,^28^ although estimates were based on only six studies. The PI further indicates that the prevalence in future studies will most likely exceed general population levels. Chronic insomnia has been under-recognised in previous research despite causing significant impairment in social, occupational, educational or behavioural functioning.^28^ However, robust estimates of sleep disturbance in IDPs in the context of armed conflicts are still lacking as most data stemmed from studies following the Great East Japan Earthquake, restricting the generalisability of findings.

PTSD rates were significantly higher among IDPs impacted by armed conflicts compared with those impacted by natural hazards. Whereas natural hazards are typically acute events of limited duration, armed conflicts often persist over long periods and are frequently still ongoing at the time of data collection, exposing many IDPs living in regional proximity to conflict zones to continued insecurity and threats to their safety. Overall, exposure to armed conflict is often associated with increased human rights violations, loss of trust in others and institutions and ongoing violence.^7^ The lack of a statistically significant difference in depression prevalence between those affected by natural hazard and armed conflicts may be attributable to key risk factors, such as persistent social disadvantages or financial strain, being prevalent among IDPs irrespective of the underlying displacement cause. The latter factor may also explain why depression was significantly higher among IDPs in low-income countries. We observed higher prevalence rates of PTSD among female IDPs, no gender differences in depression and insufficient evidence for generalised anxiety disorder. The effects are overall lower compared with gender differences in other contexts^29^ despite the frequent occurrence of gender-based violence in displacement settings.^30^ Overall, the considerable heterogeneity between studies remained largely unexplained by moderator analyses, reflecting limitations of the existing evidence base. The heterogeneity between studies was furthermore evident in the notably higher prevalence of depression in studies with gender-specific reporting compared with studies without such differentiation.

The finding that IDPs showed markedly higher rates of mental disorders than non-displaced populations suggests a significant association between displacement and mental health. This difference may reflect direct effects of displacement itself but also potential confounding factors such as differences in trauma exposure, social support or access to humanitarian services, which were not assessed in studies. Increased cumulative stressors arising from displacement, such as the loss of one’s home and social ties, exposure to additional traumatic events and ongoing adversities, can compound pre-existing vulnerabilities. In this meta-analysis, over half of IDPs were accommodated in temporary facilities, which often lack security, privacy or access to services. Previous research among displaced individuals (encompassing both IDPs and refugees) showed that people living in camps and institutionalised or temporary shelters exhibit poorer mental health compared with permanently resettled or privately accommodated individuals.^7 10^ Importantly, almost half of the IDPs in this meta-analysis were individuals from an ethnic minority and with low education, who are already at elevated risk for mental ill-health.^31^ It is important to consider that the nature of comparative studies varies widely across contexts. Whereas non-displaced individuals who experience natural hazards have most likely been only mildly exposed to these events, non-displaced residents living in conflict-settings are likely to also have experienced a range of traumatic events. Moreover, insufficient reporting often limited an assessment of the comparability of the samples with respect to demographic characteristics, socioeconomic status and other relevant variables. The consideration of covariates appears important for future studies to disentangle the independent impact of displacement experiences, particularly for depression.

### Limitations

With a total of 124 studies, our findings are based on a comprehensive dataset. However, studies in active conflict regions are often difficult to conduct due to security risks, restricted access or poor infrastructure. Consequently, some of the most severely affected areas in recent years, such as Yemen, the Democratic Republic of the Congo and Myanmar,^1^ were not represented in our dataset. Moreover, the majority of included studies were conducted in the context of armed conflict. Slow-onset environmental changes, such as desertification and prolonged droughts, have so far received little attention in mental health and displacement research, despite their expected growing importance in the future.^2^ The considerable heterogeneity across studies limits the epidemiological interpretability of our pooled estimates at a global level. Rather, they should be understood as indicative of a substantial mental health burden among IDPs that varies markedly across contexts, populations or study designs. In addition to differences in displacement cause, samples varied substantially in duration of displacement or accommodation type, while reporting of contextual factors differed markedly across studies. The overall high degree of selection bias across studies further limits the generalisability of the findings and future studies should employ sampling strategies that enhance representativeness, such as probabilistic quota sampling, particularly where random sampling is not feasible.^32^ Results from our sensitivity analyses indicate that sampling strategy and measurement instruments (ie, self-report questionnaires vs clinician-administered interviews) can affect prevalence estimates. While the observed differences were not statistically significant, it is possible that true differences were masked by the overall between-study heterogeneity. The influence of measurements on prevalence estimates has been discussed in previous literature^33^ and should be considered in future research. Finally, further research on outcomes beyond PTSD and depression, such as generalised anxiety disorder and suicidality, is urgently needed.

### Conclusions on practical implications

Although mental disorders should not be regarded as an inevitable consequence of displacement, results from this meta-analysis highlight the association between displacement and related contextual factors and mental disorders, underscoring the need for targeted mental health services for IDPs. The high prevalence of probable insomnia and its transdiagnostic characteristics suggest that sleep disturbances may serve as an early entry point for mental health screening and intervention in humanitarian settings. At the same time, including a systematic assessment of suicidal ideation into comprehensive screening protocols appears essential to ensure timely identification and effective support for at-risk individuals. Effective screening protocols may be particularly relevant for temporary resettlement sites and could be implemented at registration. The current evidence base furthermore suggests that screening priority may be given to IDPs from ethnic minorities and those with low levels of education. Nonetheless, it must be acknowledged that the results from this meta-analysis reflect broad patterns across highly heterogeneous populations and settings. Finally, IDPs have severely restricted access to international support and healthcare resources. Scalable and locally deliverable interventions are therefore urgently needed in the long term to treat or prevent chronic mental disorders and avoid further burdening already overstretched healthcare systems.^34^

## Supplementary material

10.1136/bmjgh-2025-023173 online supplemental file 1

## Data Availability

Data are available on reasonable request.

## References

[1] United Nations High Commissioner for Refugees (UNHCR) (2025). Global trends: forced displacement in 2024. https://www.unhcr.org/global-trends-report-2024.

[2] Schewel K, Dickerson S, Madson B (2024). How well can we predict climate migration? A review of forecasting models. Front Clim.

[3] International Displacement Monitoring Centre (iDMC) (2025). Global report on internal displacement 2025.

[4] Miller KE, Rasmussen A (2017). The mental health of civilians displaced by armed conflict: an ecological model of refugee distress. Epidemiol Psychiatr Sci.

[5] Schwartz RM, Rasul R, Kerath SM (2018). Displacement during Hurricane Sandy: The impact on mental health. J Emerg Manag.

[6] Tesfaye AH, Sendekie AK, Kabito GG (2024). Post-traumatic stress disorder and associated factors among internally displaced persons in Africa: A systematic review and meta-analysis. PLoS ONE.

[7] Steel Z, Chey T, Silove D (2009). Association of torture and other potentially traumatic events with mental health outcomes among populations exposed to mass conflict and displacement: a systematic review and meta-analysis. JAMA.

[8] Bedaso A, Duko B (2022). Epidemiology of depression among displaced people: A systematic review and meta-analysis. Psychiatry Res.

[9] Andualem F, Melkam M, Takelle GM (2024). Prevalence of posttraumatic stress disorder and associated factors among displaced people in Africa: a systematic review and meta-analysis. Front Psychiatry.

[10] Porter M, Haslam N (2005). Predisplacement and postdisplacement factors associated with mental health of refugees and internally displaced persons: a meta-analysis. JAMA.

[11] Blackmore R, Boyle JA, Fazel M (2020). The prevalence of mental illness in refugees and asylum seekers: A systematic review and meta-analysis. PLoS Med.

[12] Salama P, Spiegel P, Brennan R (2001). No less vulnerable: the internally displaced in humanitarian emergencies. The Lancet.

[13] Melnychuk T, Klimovskyi S, Lunov V (2019). Psychological Disorders of Internally Displaced Persons. International Journal of Advanced Biotechnology and Research.

[14] Page MJ, McKenzie JE, Bossuyt PM (2021). The PRISMA 2020 statement: an updated guideline for reporting systematic reviews. BMJ.

[15] Ouzzani M, Hammady H, Fedorowicz Z (2016). Rayyan-a web and mobile app for systematic reviews. Syst Rev.

[16] World Health Organization (WHO) (2022). International classification of diseases. https://icd.who.int.

[17] American Psychiatric Association (APA) (2022). Diagnostic and statistical manual of mental disorders.

[18] World Bank (2025). World bank country and lending groups. https://datahelpdesk.worldbank.org/knowledgebase/articles/906519.

[19] Posit

[20] Hoppen TH, Meiser-Stedman R, Kip A (2024). The efficacy of psychological interventions for adult post-traumatic stress disorder following exposure to single versus multiple traumatic events: a meta-analysis of randomised controlled trials. Lancet Psychiatry.

[21] Egger M, Davey Smith G, Schneider M (1997). Bias in meta-analysis detected by a simple, graphical test. BMJ.

[22] Sterne JAC, Sutton AJ, Ioannidis JPA (2011). Recommendations for examining and interpreting funnel plot asymmetry in meta-analyses of randomised controlled trials. BMJ.

[23] Kip A, Valencia S, Glunz E (2024). Prevalence of mental disorders in adult populations from the Global South following exposure to natural hazards: a meta-analysis. Epidemiol Psychiatr Sci.

[24] Peters JL, Sutton AJ, Jones DR (2010). Assessing Publication Bias in Meta-Analyses in the Presence of Between-Study Heterogeneity. J R Stat Soc Ser A Stat Soc.

[25] Charlson F, van Ommeren M, Flaxman A (2019). New WHO prevalence estimates of mental disorders in conflict settings: a systematic review and meta-analysis. Lancet.

[26] Hoppen TH, Priebe S, Vetter I (2021). Global burden of post-traumatic stress disorder and major depression in countries affected by war between 1989 and 2019: a systematic review and meta-analysis. BMJ Glob Health.

[27] Koenen KC, Ratanatharathorn A, Ng L (2017). Posttraumatic stress disorder in the World Mental Health Surveys. Psychol Med.

[28] van Straten A, Weinreich KJ, Fábián B (2025). The Prevalence of Insomnia Disorder in the General Population: A Meta-Analysis. J Sleep Res.

[29] Nolting IKL, Morina N, Hoppen TH (2025). A meta-analysis on gender differences in prevalence estimates of mental disorders following exposure to natural hazards. Eur J Psychotraumatol.

[30] Tadesse G, Andualem F, Rtbey G (2024). Gender-based violence and its determinants among refugees and internally displaced women in Africa: systematic review and meta-analysis. BMC Public Health.

[31] Kirkbride JB, Anglin DM, Colman I (2024). The social determinants of mental health and disorder: evidence, prevention and recommendations. World Psychiatry.

[32] Kip A, Kurt G, Acartürk C (2025). Reflections on mental health research in post-disaster settings. PLOS Glob Public Health.

[33] Hyland P, Shevlin M (2024). Clinician-administered interviews should not be considered the ‘gold standard’ method of assessing psychological distress. New Ideas Psychol.

[34] Schäfer SK, Thomas LM, Lindner S (2023). World Health Organization’s low-intensity psychosocial interventions: a systematic review and meta-analysis of the effects of Problem Management Plus and Step-by-Step. World Psychiatry.

