# Supplementary material for: Mental health of internally displaced persons: a meta-analysis
Source: BMJ Glob Health. 2026 Jun 4;11(6):e023173. doi: 10.1136/bmjgh-2025-023173 (PMC13239451; doi:10.1136/bmjgh-2025-023173)
Supplement: online supplemental file 1 [file bmjgh-11-6-s001.pdf]

Mental health of internally displaced individuals – a meta-analysis

Supplementary Material

Supplement 1: Search strategy 1

Supplement 2: References of related reviews considered for study identification 3

Supplement 3: Characteristics of included studies 6

Supplement 4: Reference list of included studies 18

Supplement 5: List of excluded studies with reasons for exclusion 31

Supplement 6: Results of moderator and subgroup analyses 49

Supplement 7: Overview of mental disorders investigated in <4 studies 50

Supplement 8: Results for publication bias and sensitivity analyses 51

Supplement 9: Reported adjusted odds ratio for comparisons IPD vs. non-displaced 53

Supplement 10: Definition of displacement vs. non-displacement status in comparative studies 55

Supplement 1: Search strategy

Search 1 inception until 02/10/2024
Medline/PsycINFO: 14.775
Web of Science: 13.975

Update search prior to final analyses 20/06/2025
Medline/PsycINFO: 745
Web of Science: 681

AND

	**Concept 1**	**Concept 2**
**Key Terms**<br>all fields for Concept 1<br><br>title/abstract/ key words for concept 2	displace* OR evacu* OR resettle* OR relocat* OR "temporary ho*" OR "temporary accommodation" OR "relief camp" OR "tent village" OR shelter	"mental disorder*" OR "mental health" OR "mental disease*" OR "mental illness*"<br>OR "affective disorder*" OR "mood disorder*" OR depress* OR bipolar OR mania<br>OR ptsd OR "posttraumatic stress disorder" OR "post-traumatic stress disorder" OR "acute stress"<br>OR grief OR bereav*<br>OR anxiety OR phobia OR panic<br>OR psychosis OR schizophre*<br>OR "substance use disorder*" OR "alcohol dependen*" OR "drug dependen*" OR alcoholism OR addiction<br>OR "eating disorder*" OR "sleep disorder*" OR insomnia OR "suicid*" OR somatoform OR "obsessive compulsive disorder*" OR OCD OR "personality disorder*"

MEDLINE/PsyINFO:
TI (“mental disorder*“ OR “mental health” OR “mental disease*” OR “mental illness*” OR “affective disorder*” OR “mood disorder*” OR depress* OR bipolar OR mania OR ptsd OR “posttraumatic stress disorder” OR “post-traumatic stress disorder” OR “acute stress” OR grief OR bereav* OR anxiety OR phobia OR panic OR psychosis OR schizophre* OR “substance use disorder*” OR “alcohol dependen*” OR “drug dependen*” OR alcoholism OR addiction OR “eating disorder*” OR “sleep disorder*” OR insomnia OR “suicid*” OR somatoform OR “obsessive compulsive disorder*” OR OCD OR “personality disorder*”) OR AB (“mental disorder*“ OR “mental health” OR “mental disease*” OR “mental illness*” OR “affective disorder*” OR “mood disorder*” OR depress* OR bipolar OR mania OR ptsd OR “posttraumatic stress disorder” OR “post-traumatic stress disorder” OR “acute stress” OR grief OR bereav* OR anxiety OR phobia OR panic OR psychosis OR schizophre* OR “substance use disorder*” OR “alcohol dependen*” OR “drug dependen*” OR alcoholism OR addiction OR “eating disorder*” OR “sleep disorder*” OR insomnia OR “suicid*” OR somatoform OR “obsessive compulsive disorder*” OR OCD OR “personality disorder*”) OR SU (“mental disorder*“ OR “mental health” OR “mental disease*” OR “mental illness*” OR “affective disorder*” OR “mood disorder*” OR depress* OR bipolar OR mania OR ptsd OR “posttraumatic stress disorder” OR “post-traumatic stress disorder” OR “acute stress” OR grief OR bereav* OR anxiety OR phobia OR panic OR psychosis OR schizophre* OR “substance use disorder*” OR “alcohol dependen*” OR “drug dependen*” OR alcoholism OR addiction OR “eating disorder*” OR “sleep disorder*” OR insomnia OR “suicid*” OR somatoform OR “obsessive compulsive disorder*” OR OCD OR “personality disorder*”)
AND
(TX displace* OR evacu* OR resettle* OR relocat* OR “temporary ho*” OR “temporary accommodation” OR “relief camp“ OR “tent village“ OR shelter)
Filter: Peer-Reviewer journal articles

Web of Science:
((((TI=(“mental disorder*“ OR “mental health” OR “mental disease*” OR “mental illness*” OR “affective disorder*” OR “mood disorder*” OR depress* OR bipolar OR mania OR ptsd OR “posttraumatic stress disorder” OR “post-traumatic stress disorder” OR “acute stress” OR grief OR bereav* OR anxiety OR phobia OR panic OR psychosis OR schizophre* OR “substance use disorder*” OR “alcohol dependen*” OR “drug dependen*” OR alcoholism OR addiction OR “eating disorder*” OR “sleep disorder*” OR insomnia OR “suicid*” OR somatoform OR “obsessive compulsive disorder*” OR OCD OR “personality disorder*”)) OR AB=(“mental disorder*“ OR “mental health” OR “mental disease*” OR “mental illness*” OR “affective disorder*” OR “mood disorder*” OR depress* OR bipolar OR mania OR ptsd OR “posttraumatic stress disorder” OR “post-traumatic stress disorder” OR “acute stress” OR grief OR bereav* OR anxiety OR phobia OR panic OR psychosis OR schizophre* OR “substance use disorder*” OR “alcohol dependen*” OR “drug dependen*” OR alcoholism OR addiction OR “eating disorder*” OR “sleep disorder*” OR insomnia OR “suicid*” OR somatoform OR “obsessive compulsive disorder*” OR OCD OR “personality disorder*”)) OR AK=(“mental disorder*“ OR “mental health” OR “mental disease*” OR “mental illness*” OR “affective disorder*” OR “mood disorder*” OR depress* OR bipolar OR mania OR ptsd OR “posttraumatic stress disorder” OR “post-traumatic stress disorder” OR “acute stress” OR grief OR bereav* OR anxiety OR phobia OR panic OR psychosis OR schizophre* OR “substance use disorder*” OR “alcohol dependen*” OR “drug dependen*” OR alcoholism OR addiction OR “eating disorder*” OR “sleep disorder*” OR insomnia OR “suicid*” OR somatoform OR “obsessive compulsive disorder*” OR OCD OR “personality disorder*”)))
AND
ALL=(displace* OR evacu* OR resettle* OR relocat* OR “temporary ho*” OR “temporary accommodation” OR “relief camp“ OR “tent village“ OR shelter)

Supplement 2: References of related reviews considered for study identification

Abbara, A., Rayes, D., Ekzayez, A., Jabbour, S., Marzouk, M., Alnahhas, H., ... & Fouad, F. M. (2022). The health of internally displaced people in Syria: are current systems fit for purpose? *Journal of Migration and Health*, *6*, 100126. https://doi.org/10.1016/j.jmh.2022.100126

Ahmed, S. H., Zakai, A., Zahid, M., Jawad, M. Y., Fu, R., & Chaiton, M. (2024). Prevalence of post-traumatic stress disorder and depressive symptoms among civilians residing in armed conflict-affected regions: a systematic review and meta-analysis. *General Psychiatry*, *37*(3), e101438. https://doi.org/10.1136/gpsych-2023-101438

Alhaffar, M. H. D. B. A. & Janos, S. (2021). Public health consequences after ten years of the Syrian crisis: A literature review. *Globalization And Health*, *17*(1), 111. https://doi.org/10.1186/s12992-021-00762-9

Amodu, O. C., Richter, M. S. & Salami, B. O. (2020). A Scoping Review of the Health of Conflict-Induced Internally Displaced Women in Africa. *International Journal Of Environmental Research And Public Health*, *17*(4), 1280. https://doi.org/10.3390/ijerph17041280

Andualem, F., Melkam, M., Takelle, G. M., Nakie, G., Tinsae, T., Fentahun, S., ... & Godana, T. N. (2024). Prevalence of posttraumatic stress disorder and associated factors among displaced people in Africa: a systematic review and meta-analysis. *Frontiers in psychiatry*, *15*, 1336665. https://doi.org/10.3389/fpsyt.2024.1336665

Bedaso, A. & Duko, B. (2022). Epidemiology of depression among displaced people: A systematic review and meta-analysis. *Psychiatry Research*, *311*, 114493. https://doi.org/10.1016/j.psychres.2022.114493

Böcker, A. & Hunter, A. (2022). Older Refugees and Internally Displaced People in African Countries: Findings from a Scoping Review of Literature. *Journal Of Refugee Studies*. https://doi.org/10.1093/jrs/feac023

Bogic, M., Njoku, A. & Priebe, S. (2015). Long-term mental health of war-refugees: A systematic literature review. *BMC International Health And Human Rights*, *15*(1). https://doi.org/10.1186/s12914-015-0064-9

Charlson, F., van Ommeren, M., Flaxman, A., Cornett, J., Whiteford, H., & Saxena, S. (2019). New WHO prevalence estimates of mental disorders in conflict settings: a systematic review and meta-analysis. *The Lancet*, *394*(10194), 240-248. https://doi.org/10.1016/S0140-6736(19)30934-1

Hanáková, K., Lindberg, L. G. & Carlsson, J. (2024). Sex differences in trauma exposure and PTSD symptomatology among refugees, internally displaced people, and asylum seekers: A systematic

literature review. *Psychiatry Research*, *339*, 116014. https://doi.org/10.1016/j.psychres.2024.116014

Jang, S., Ekyalongo, Y., & Kim, H. (2021). Systematic review of displacement and health impact from natural disasters in Southeast Asia. *Disaster medicine and public health preparedness*, *15*(1), 105-114. https://doi.org/10.1017/dmp.2019.125

Marie, M., SaadAdeen, S. & Battat, M. (2020). Anxiety disorders and PTSD in Palestine: A literature review. *BMC Psychiatry*, *20*(1). https://doi.org/10.1186/s12888-020-02911-7

Mellor, R., Werner, A., Moussa, B., Mohsin, M., Jayasuriya, R., & Tay, A. K. (2021). Prevalence, predictors and associations of complex post-traumatic stress disorder with common mental disorders in refugees and forcibly displaced populations: a systematic review. *European Journal of Psychotraumatology*, *12*(1), 1863579. https://doi.org/10.1080/20008198.2020.1863579

Melnychuk, T., Klimovskyi, S., & Lunov, V. (2019). Psychological disorders of internally displaced persons. *International Journal of Advanced Biotechnology and Research,* (1), 525–537.

Mohammadi, M., Jafari, H., Etemadi, M., Dalugoda, Y., Ali, H. M., Phung, H., Ahmadvand, A., Dwirahmadi, F., Barnes, P. & Chu, C. (2023). Health Problems of Increasing Man-Made and Climate-Related Disasters on Forcibly Displaced populations: A Scoping Review on Global Evidence. *Disaster Medicine And Public Health Preparedness*, *17*. https://doi.org/10.1017/dmp.2023.159

Morina, N., Akhtar, A., Barth, J. & Schnyder, U. (2018). Psychiatric Disorders in Refugees and Internally Displaced Persons After Forced Displacement: A Systematic Review. *Frontiers in Psychiatry*, *9*. https://doi.org/10.3389/fpsyt.2018.00433

Peevey, N., Flores, E. & Seguin, M. (2022). Common mental disorders and coping strategies amongst internally displaced Colombians: A systematic review. *Global Public Health*, *17*(12), 3440–3454. https://doi.org/10.1080/17441692.2022.2049343

Porter, M. & Haslam, N. (2005). Predisplacement and Postdisplacement Factors Associated With Mental Health of Refugees and Internally Displaced Persons. *JAMA*, *294*(5), 602–612. https://doi.org/10.1001/jama.294.5.602

Quosh, C., Eloul, L., & Ajlani, R. (2013). Mental health of refugees and displaced persons in Syria and surrounding countries: A systematic review. *Intervention Journal of Mental Health and Psychosocial Support in Conflict Affected Areas*, *11*(3), 276–294.

Sisenop, F., Chatarajupalli, P., Bain, P. A., Kaade, H., & Lindert, J. (2025). Human rights violations are associated with forcibly displaced population's mental health - a systematic review and meta-analysis. *Frontiers in Public Health*, *12*, 1454331. https://doi.org/10.3389/fpubh.2024.1454331

Steel, Z., Chey, T., Silove, D., Marnane, C., Bryant, R. A. & Van Ommeren, M. (2009). Association of Torture and Other Potentially Traumatic Events With Mental Health Outcomes Among Populations Exposed to Mass Conflict and Displacement. *JAMA*, *302*(5), 537–549. https://doi.org/10.1001/jama.2009.1132

Tesfaye, A. H., Sendekie, A. K., Kabito, G. G., Engdaw, G. T., Argaw, G. S., Desye, B., Angelo, A. A., Aragaw, F. M. & Abere, G. (2024). Post-traumatic stress disorder and associated factors among internally displaced persons in Africa: A systematic review and meta-analysis. *PLoS ONE*, *19*(4), e0300894. https://doi.org/10.1371/journal.pone.0300894

Weaver, H., & Roberts, B. (2010). Drinking and displacement: a systematic review of the influence of forced displacement on harmful alcohol use. *Substance Use & Misuse*, *45*(13), 2340-2355. https://doi.org/10.3109/10826081003793920

Supplement 3: Characteristics of included studies

Studies with internally displaced sample only

Study	Location	Displacement cause	Stage of displacement cause	Time since onset (months)	*N*	Mean age (SD)	% female	Outcome (instrument)
**Abiama et al., 2022**	Nigeria	armed conflict	ongoing	76	1059	34.3 (13.7)	45.2	PTSD (PCL-5)
**Abukar et al., 2025**	Somalia	mixed	ongoing	n.r.	364	n.r.	70.3	PTSD (PCL-5)
**Adhikari Baral & K.C., 2019**	Nepal	natural hazard	past	10	245	n.r.	n.r.	PTSD (PCL-5)
**Aldabbour et al., 2024**	West Bank	armed conflict	ongoing	8	339	n.r.	59.9	PTSD (PCL-5)
**Aldabbour et al., 2025**	West Bank	armed conflict	ongoing	13	952	32.8 (12.5)	62.0	PTSD (PCL-5)<br>GAD (GAD-7)<br>MDD (PHQ-9)
**Al Ibraheem et al., 2017**	Syria	armed conflict	ongoing	60	195	37.0 (12.6)	n.r.	PTSD (CAPS-2)
**Alejo et al., 2007**	Colombia	armed conflict	ongoing	mixed	851	37.4 (14.0)	62.7	PTSD (CAPS)
**Ali et al., 2023**	Somalia	mixed	ongoing	360	401	n.r.	83.3	PTSD (HTQ)
**Aluh et al., 2024**	Nigeria	armed conflict	ongoing	147	520	33.5 (13.5)	76.2	SUD (DUDIT)
**Awa et al., 2024**	Nigeria	armed conflict	ongoing	552	486	37.0 (11.3)	53.3	PTSD (HTQ-5)
**Başoğlu et al., 2002**	Türkiye	natural hazard	ongoing	7.7	1000	34.5 (13.3)	68.0	PTSD (TSSC)
**Bekeko et al., 2025**	Ethiopia	armed conflict	past	53	997	45.4 (12.7)	52.8	MDD (PHQ-9)<br>GAD (GAD-7)
**Cheung et al., 2019**	Ukraine	armed conflict	ongoing	24	2203	45.1 (n.r.)	68.0	GAD (GAD-7)<br>MDD (PHQ-9)
**Chukwuorji et al., 2017**	Nigeria	armed conflict	ongoing	n.r.	859	38.8 (1.9)	49.4	PTSD (HTQ)
**Cofini et al., 2015**	Italy	natural hazard	past	16.5	271	43.0 (16.0)	54.2	PTSD (DTS)
**Echenique et al., 2008**	Colombia	armed conflict	ongoing	n.r.	202	n.r.	n.r.	PTSD (TQ)

**Espinoza-Neyra et al., 2017**	Peru	natural hazard	ongoing	1.5	121	n.r.	n.r.	PTSD (DTS)
**Farina et al., 2012**	Italy	natural hazard	past	21	300	n.r.	66.7	Insomnia (ISI)
**Faronbi et al., 2020**	Nigeria	armed conflict	ongoing	n.r.	240	34.5 (11.1)	58.8	PTSD (PCL-C)
**Freh & North, 2025**	Iraq	armed conflict	past	113	215	27.9 (6.0)	100.0	PTSD (PCL-5)
**Garabiles et al., 2023**	Philippines	natural hazard	past	n.r.	622	33.8 (11.7)	45.6	PTSD (PCL-5)
		armed conflict	past		424	51.0 (12.6)	61.1	
		natural hazard	past		523	28.5 (12.6)	60.6	
**Gebreyesus et al., 2024a**	Ethiopia	armed conflict	ongoing	9	2071	n.r.	55.0	PTSD (PCL-C)
**Gebreyesus et al., 2024b**	Ethiopia	armed conflict	ongoing	9	1965	n.r.	55.1	MDD (PHQ-9)
**Ghaffari-Nejad et al., 2007**	Iran	natural hazard	past	n.r.	400	37.8 (12.7)	47.3	PGD (ICG)
**Hagh-Shenas et al., 2006**	Iran	natural hazard	past	1.4	145	31.8 (n.r.)	57.2	PTSD (PSS-I)
**Hamid & Musa, 2010**	Sudan	armed conflict	ongoing	24	430	34.6 (13.9)	49.3	PTSD (PCL-C)
**Hashmi et al., 2011**	Pakistan	natural hazard	past	6	361	35.0 (13.8)	57.3	PTSD (PCL-S)
**Heeke et al., 2015**	Colombia	armed conflict	ongoing	579	295	48.6 (n.r.)	61.4	PTSD (PCL-C) PGD (PG-13)
**Hemmeda et al., 2025**	Sudan	armed conflict	ongoing	5	1470	35.3 (13.9)	81.4	PTSD (HTQ)
**Hodges et al., 2023**	United States	natural hazard	past	42	319	38.7 (12.1)	71.2	PTSD (PCL-C)
**Howard et al., 1999**	Philippines	natural hazard	past	72	351	n.r.	n.r.	MDD, GAD, Dysthymia, Panic disorder, Bipolar disorder (PRIME-MD) PTSD (PCL-S)
**Ibrahim et al., 2018**	Iraq	armed conflict	ongoing	35	416	31.7 (12.6)	100.0	PTSD (PCL-5)
**Ide-Okochi et al., 2022**	Japan	natural hazard	past	53	8330	62.3 (17.3)	56.7	Insomnia (AIS)

**Jann et al., 2024**	Iraq	armed conflict	past	55	199	41.4 (13.0)	53.3	PTSD (PCL-5)<br>PGD (ICG)
**Khalil et al., 2024**	Sudan	armed conflict	ongoing	9	297	n.r.	74.7	PTSD (PCL-5)
**Kim et al., 2007**	Sudan	armed conflict	ongoing	23	1253	34.0 (0.3)	100	MDD (PHQ-9)
**King et al., 2016**	United States	natural hazard	past	4.8	541	41.3 (14.7)	59.1	PTSD, MDD (DIS-4)
**Kira et al., 2023**	Syria	armed conflict	ongoing	127	891	30.8 (12.0)	46.5	PTSD, cPTSD (ITQ)<br>MDD (PHQ-9)<br>GAD (GAD-7)
**Kozarić-Kovacić et al., 2000**	Croatia	armed conflict	past	n.r.	368	38.9 (n.r.)	57.3	PTSD (Watson's PTSD interview)
**Kumar et al., 2007**	India	natural hazard	past	2	314	35.8 (14.0)	51.3	PTSD (HTQ)
**Kuo et al., 2007**	Taiwan	natural hazard	past	12	272	46.5 (18.4)	56.3	PTSD (DTS)
**Larrance et al., 2007**	United States	natural hazard	past	8.2	366	45.9 (15.4)	53.3	MDD (PHQ-9)
**Liu et al., 2012**	China	natural hazard	past	6	9556	44.5 (n.r.)	54.7	PTSD (PCL-C)
**Ma et al., 2010**	China	natural hazard	n.r.	n.r.	182	44.7 (11.4)	64.8	PTSD (SCID-P, MINI)
**Ma et al., 2013**	China	natural hazard	past	9	151	44.4 (14.9)	45.0	MDD, Suicidality (MINI)<br>PTSD (Breslau Scale)
**Madoro et al., 2020**	Ethiopia	armed conflict	past	12	625	33.0 (11.2)	47.5	PTSD (PCL-5)
**Makango et al., 2023**	Ethiopia	armed conflict	ongoing	13.5	406	39.1 (14.6)	51.7	PTSD (PCL-5)
**Makhashvili et al., 2014**	Georgia	armed conflict	past	239<br>39	1193<br>996	n.r.	65.3<br>66.8	MDD (PHQ-9)<br>GAD (GAD-7)
**Mamed et al., 2025**	Ethiopia	armed conflict	ongoing	56	367	n.r.	44.7	PTSD (PCL-C)
**Manafe et al., 2024**	Mozambique	mixed	ongoing	65	748	n.r.	65.1	MDD (PHQ-9)<br>GAD (GAD-7)
**McGinty et al., 2023**	Ukraine	armed conflict	ongoing	24	2198	45.0 (17.0)	68.1	PTSD, cPTSD (ITQ)

**Melese et al., 2024**	Ethiopia	armed conflict	past	36	410	33.0 (1.7)	38.0	PTSD (PCL-5)<br>MDD (PHQ-9)
**Melkam et al., 2025**	Ethiopia	armed conflict	ongoing	50	398	37.0 (13.1)	82.4	PTSD (PCL-5)
**Mirkhan Ahmed et al., 2024**	Iraq	armed conflict	ongoing	n.r.	622	n.r.	100.0	MDD (PHQ-9)<br>GAD (GAD-7)
**Mohamed & Kehir, 2024**	Sudan	armed conflict	ongoing	4	143	n.r.	86.0	PTSD (PCL-C)<br>MDD (PHQ-9)
**Montero-Zamora et al., 2023**	United States	natural hazard	past	48	319	38.5 (12.0)	70.8	PTSD (PCL-C)<br>GAD (GAD-7)
**Muhummed et al., 2024**	Ethiopia	mixed	ongoing	n.r.	404	n.r.	52.0	PTSD (PCL-5)
**Muhtz et al., 2011**	Germany	armed conflict	past	n.r.	502	71.0 (n.r.)	55.6	PTSD (PDS)
**Mumin et al., 2022**	Somalia	mixed	ongoing	n.r.	585	n.r.	56.1	MDD (PHQ-9)
**Naeem et al., 2011**	Pakistan	natural hazard	past	18	1291	32.9 (n.r.)	60.3	PTSD (TSSC)
**Pabón-Poches et al., 2024**	Colombia	armed conflict	ongoing	n.r.	1368	39.7 (15.4)	62.6	PTSD (EGEP-5)
**Pinchuk et al., 2024**	Ukraine	armed conflict	ongoing	3	520	n.r.	n.r.	MDD (PHQ-9)<br>Insomnia (ISI)
**Polyvianaia et al., 2025**	Ukraine	armed conflict	ongoing	20	436	n.r.	n.r.	MDD (PHQ-9)<br>GAD (GAD-7)
**Rajkumari et al., 2024**	India	armed conflict	ongoing	6	310	39.3 (15.2)	57.4	PTSD (PCL-5)<br>GAD (GAD-7)
**Ramachandran et al., 2019**	Ukraine	armed conflict	ongoing	24	2203	n.r.	67.0	AUD (AUDIT)
**Rana et al., 2008**	Pakistan	natural hazard	past	7	111	n.r.	50.5	PTSD (TSSC)
**Ranasinghe & Levy, 2007**	Sri Lanka	natural hazard	past	6	264	38.4 (14.5)	61.0	PTSD (PSS-I)
**Richa et al., 2020**	Iraq	armed conflict	ongoing	28	150	36.0 (n.r.)	50.7	PTSD (MINI)
**Richards et al., 2011**	Colombia	armed conflict	ongoing	528	103	37.8 (14.3)	63.1	PTSD (Piñeda's PTSD Checklist)
**Roberts et al., 2008**	Uganda	armed conflict	past	250	1210	35.3 (n.r.)	60.1	PTSD (HTQ)
**Roberts et al., 2011**	Uganda	armed conflict	past	250	1206	35.0 (n.r.)	60.0	AUD (AUDIT)
**Robinson et al., 2025**	Colombia	armed conflict	ongoing	701	498	n.r.	n.r.	PTSD (PCL-5)

**Salah et al., 2013**	Sudan	armed conflict	past	300	1876	n.r.	55.7	MDD, Dysthymia, Suicidality, Hypomanic episode, Panic disorder, Agoraphobia, Social phobia, OCD, PTSD, AUD, SUD, Psychotic disorder, GAD, Antisocial personality disorder (MINI)
**Şalcioğlu et al., 2003**	Türkiye	natural hazard	ongoing	20	586	38.1 (14.6)	57.5	PTSD (TSSC)
**Şalcioğlu et al., 2007**	Türkiye	natural hazard	past	43.5	769	36.1 (13.3)	65.9	PTSD (TSSC)
**Şalcioğlu et al., 2018**	Türkiye	natural hazard	past	16.5	150<br>182	34.7 (11.2)<br>32.2 (11.3)	74.0<br>43.4	PTSD (TSSC)
**Shehab et al., 2008**	United States	natural hazard	past	25	603	44.7 (0.9)	51.6	MDD (PHQ-9)
**Shevchenko et al., 2020**	Ukraine	armed conflict	ongoing	60	212	n.r.	n.r.	MDD (PHQ-9)<br>GAD (GAD-7)
**Sinisterra Mosquera et al., 2010**	Colombia	armed conflict	ongoing	n.r.	110	38.5 (13.4)	65.5	PTSD (Piñeda's PTSD Checklist)
**Siriwardhana et al., 2015**	Sri Lanka	armed conflict	past	264	324	38.5 (n.r.)	65.4	MDD (PRIME-MD)<br>PTSD (CIDI)
**Spell et al., 2008**	United States	natural hazard	past	5	260	39.0 (n.r.)	100.0	PTSD (PDS)
**Sprang et al., 2009**	United States	natural hazard	past	12	101	42.0 (n.r.)	62.4	PTSD (PCL-C)
**Suzuki et al., 2021**	Japan	natural hazard	past	68	710	53.8 (n.r.)	55.8	Insomnia (AIS)
**Tadesse et al., 2024**	Ethiopia	armed conflict	ongoing	19	751	34.3 (10.0)	67.1	MDD (PHQ-9)<br>PTSD (PCL-5)<br>GAD (GAD-7)

**Taha & Sijbrandij, 2021**	Iraq	armed conflict	ongoing	12	822	n.r.	43.6	PTSD (HTQ)
**Tekeli-Yesil et al., 2018**	Syria	armed conflict	ongoing	74	244	35.8 (10.2)	73.0	MDD, Suicidality, Panic disorder, Agoraphobia, PTSD, GAD (MINI)
**Thapa & Hauff, 2005**	Nepal	armed conflict	ongoing	88	290	40.9 (14.2)	39.0	PTSD (PCL-C)
**Timmer et al., 2023**	Ukraine	armed conflict	ongoing	36	300	40.9 (14.5)	39.3	MDD (PHQ-9)
**Tural et al., 2004**	Türkiye	natural hazard	past	8	910	36.1 (13.3)	63.7	PTSD (ADAA PTSD self-test)
**Valenti et al., 2013**	Italy	natural hazard	past	38	116	43.5 (10.9)	52.6	PTSD (CAPS) MDD (BDI)
**Wang et al., 2009**	China	natural hazard	ongoing	3	622	41.0 (15.4)	55.3	PTSD (LASC)
					941	43.2 (16.9)	55.7	
**Yilmaz & Erdem, 2025**	Türkiye	natural hazard	past	9	400	38.0 (14.4)	53.0	PTSD (PCL-5) MDD (BDI)
**Zhang et al., 2011**	China	natural hazard	past	13	1181	47.3 (15.5)	62.7	PTSD (PCL-C)
**Zhang et al., 2012**	China	natural hazard	past	13	870	n.r.	53.4	PTSD (PCL-C)
				3.5	505	32.6 (12.4)	46.5	
**Zrineh et al., 2025**	West Bank	armed conflict	past	912	404	n.r.	48.0	PTSD (PCL-5)

Studies with internally displaced and non-displaced control sample

Study	Location	Displacement cause	Stage of displacement cause	Time since onset (months)	*N*	Mean age (SD)	% female	Outcome (instrument)
**Ali et al., 2012**								
Displaced	Pakistan	natural hazard	past	31	169	all: 37.7 (14.0)	all: 39.3	PTSD (DTS)
Non-displaced					131			
**Amsalem et al., 2025**								
Displaced	Israel	armed conflict	ongoing	4	243	all: 31.3 (5.6)	all: 53.8	PTSD (PHQ-9)<br>GAD (GAD-7)
Non-displaced					809			
**Asnakew et al., 2019**								
Displaced	Ethiopia	other (garbage landslide)	past	15	240	all: 33.0 (12.0)	all: 59.2	PTSD (PCL-C)
Non-displaced					590			
**Banal et al., 2010**								
Displaced	India	armed conflict	ongoing	n.r.	600	n.r.	all: 50.0	MDD, PTSD, GAD, Dysthymia, Hypomanic episode, Manic episode, Panic disorder, Agoraphobia, Social phobia, OCD, AUD, SUD, Psychotic disorder, AN, BN, Suicidality (MINI)
Non-displaced					200			
**Ben-Ezra et al., 2023**								
Displaced	Ukraine	armed conflict	ongoing	1.5	389	all: 37.2 (9.2)	all: 51.3	PTSD (ITQ)
Non-displaced					1455			
**Cerdá et al., 2013**								
Displaced	Haiti	natural hazard	past	2.75	522	n.r.	71.1	PTSD (PLC-C)<br>MDD (PHQ-9)
Non-displaced					522		66.3	

**Elhadi et al., 2022**								
Displaced	Libya	armed conflict	ongoing	111	1256	n.r.	56.4	MDD (PHQ-9) GAD (GAD-7)
Non-displaced					30301		66.2	
**Freitag et al., 2011**								
Displaced	Germany	armed conflict	past	n.r.	207	all: 75.3 (5.9)	all: 36.0	Quality of Life (EUROHIS-QOL)
Non-displaced					212			
**Freitag et al., 2013**								
Displaced	Germany	armed conflict	past	824	206	n.r.	57.3	PTSD (PDS) MDD (PHQ-9) Somatic symptoms (PHQ-15) Quality of life – physical health/mental health (SF-12v2)
Non-displaced					1451		53.0	
**Greene-Cramer et al., 2020**								
Displaced	Ukraine	armed conflict	ongoing	51	704	47.3 (13.7)	73.2	PTSD (HTQ)
Non-displaced					696	55.9 (15.5)	67.0	
**Hall et al., 2019**								
Displaced	China	natural hazard	past	1	211	all: 20.0 (2.6)	all: 66.2	PTSD (PCL-5)
Non-displaced					1665			
**Hamama et al. (2025)**								
Displaced	Israel	armed conflict	ongoing	7	221	41.0 (13.2)	69.7	MDD, GAD (PHQ-4) Health-related quality of life (SF-12) Self-mastery (Self-Mastery Scale) Meaning in life (MLQ)
Non-displaced					145	40.8 (14.0)	52.4	

**Jayasuriya, 2014**								
Displaced	Sri Lanka	armed conflict	past	348	1505	44.0 (6.2)	58.9	PTG (PTGI)
Non-displaced					955	41.0 (8.0)	55.2	
**Johnson et al., 2022**								
Displaced	Ukraine	armed conflict	ongoing	37	300	40.9 (14.5)	62.7	PTSD (PSS-I-5)
Non-displaced					1247	45.4 (17.3)	55.3	
**Kakaje et al., 2021**								
Displaced	Syria	armed conflict	ongoing	96	974	n.r.	all: 73.0	PTSD (SPTSS) Psychological distress (K10)
Non-displaced					977			
**Karatzias et al., 2023**								
Displaced	Ukraine	armed conflict	ongoing	102	501	all: 37.7 (8.2)	all: 57.1	PTSD, cPTSD (ITQ)
Non-displaced					1503			
**Kılıç et al., 2006**								
Displaced	Türkiye	natural hazard	past	48	421	all: 38.9 (16.3)	55.6	PTSD (TSSC)
Non-displaced					105		60.0	
**Kun et al., 2009**								
Displaced	China	natural hazard	past	3	478	n.r.	all: 49.1	PTSD (HTQ)
Non-displaced					524			
**Kuwert et al., 2009**								
Displaced	Germany	armed conflict	past	n.r.	239	70.6 (6.7)	47.7	GAD (GAD-7) Life satisfaction (FLZ^{M}) Resilience (RS-11)
Non-displaced					1274	69.7 (6.7)	54.2	
**Lê et al., 2013**								
Displaced	United States	natural hazard	past	21	209	n.r.	62.7	MDD (PHQ-9)
Non-displaced					499		62.5	
**Lopes Cardozo et al., 2000**	Kosovo	armed conflict	past	18.5	326	n.r.	all: 62.3	PTSD (HTQ) Social functioning (MOS-20)
Displaced					231			
Non-displaced								

								General distress (GHQ-28)
**Lushchak et al., 2023**								
Displaced	Ukraine	armed conflict	ongoing	106	505	38.5 (9.5)	86.5	PTSD (PCL-5)<br>GAD (GAD-7)<br>Stress (PSS-10)
Non-displaced					1954	37.1 (11.2)	83.2	
**Matsuoka et al., 2023**								
Displaced	Japan	natural hazard	ongoing	7	127	n.r.	63.0	MDD (SQD-D),<br>PTSD (SQD-P)
Non-displaced					528		53.2	
**Matthews et al., 2019**								
Displaced	Australia	natural hazard	past	6	313	n.r.	all: 68.8	GAD (GAD-2)<br>MDD (PHQ-2)<br>PTSD (PCL-6)
Non-displaced					1827			
**Monsalve et al., 2022**								
Displaced	Colombia	armed conflict	past	n.r.	845	n.r.	52.1	General distress (SRQ)
Non-displaced					732		52.5	
**Peleg & Gendelman, 2025**	Israel	armed conflict	ongoing	4	244	all: 41.4 (14.5)	all: 75.5	GAD (GAD-2)<br>MDD (PHQ-2)<br>PTSD (PCL-5)
Displaced								
Non-displaced					446			
**Roitblat et al., 2024**								
Displaced	Ukraine	armed conflict	ongoing	2	912	n.r.	n.r.	GAD (GAD-7)<br>Anxiety (RAR-6)
Non-displaced					431			
**Scaramutti et al., 2019**								
Displaced	United States	natural hazard	n.r.	n.r.	101	n.r.	n.r.	GAD (GAD-7)<br>PTSD (PCL-C)<br>MDD (CES-D)
Non-displaced					110			
**Shiga et al., 2021**								
Displaced	Japan	mixed	past	18	28341	n.r.	n.r.	PTSD (PCL-S)
Non-displaced					18289			
**Strauss et al., 2011**								
Displaced	Germany	armed conflict	past	n.r.	188	74.6 (5.6)	67.6	MDD (PHQ-9)
Non-displaced					213	75.9 (6.3)	62.4	

**Takahashi et al., 2016**								
Displaced	Japan	natural hazard	past	6	3160	61.2	62.0	Insomnia (AIS)
Non-displaced					3368	63.4	62.0	
**Tiyuri et al., 2023**								
Displaced	Iran	natural hazard	past	5	585	n.r.	all: 80.8	MDD (PHQ-9)
Non-displaced					1086			
**Tsuchiya et al., 2019**								
Displaced	Japan	natural hazard	past	4.5	997	n.r.	52.0	Insomnia (AIS)
Non-displaced					570		53.9	
**van Griensven et al., 2006**								
Displaced	Thailand	natural hazard	past	2, 9	371	39.5 (15.0)	59.3	PTSD (HTQ)
Non-displaced					690	42.0 (15.1)	62.5	
**Wani et al., 2020**								
Displaced	India	natural hazard	past	3	235	all: 40.7 (10.0)	all: 65.8	PTSD (PCL-C)
Non-displaced					265			
**Wen et al., 2012**								
Displaced	China	natural hazard	past	36	319	all: 47.2 (16.5)	all: 53.1	PTSD (PCL-C)
Non-displaced					2200			
**Zasiekina et al., 2023**								
Displaced	Ukraine	armed conflict	ongoing	108	567	all: 34.0 (11.0)	all: 82.6	PTSD (PCL-5)
Non-displaced					682			

Note. ADAA = Anxiety and Depression Association of America; AIS = Athens Insomnia Scale; AUD = alcohol use disorder; AUDIT = Alcohol Use Disorders Identification Test; BDI = Beck's Depression Inventory; CAPS = Clinician-Administered PTSD Scale; CES-D = Center for Epidemiologic Studies Depression Scale; CIDI = Composite International Diagnostic Interview; DIS-4 = Diagnostic Interview Schedule – Version 4; DTS = Davidson Trauma Scale; DUDIT = Drug Use Disorders Identification Test; EGEP-5 = Escala Global de Estrés Postraumático; EUROHIS-QOL = European Health Interview Survey - Quality of Life; FLZ^{M} = Questions on Life Satisfaction^{Modules}; GAD/GAD-2/GAD-7 = Generalized Anxiety Disorder (2- and 7-item scale); GHQ-28 = General Health Questionnaire – 28; HTQ/HTQ-5 = Harvard Trauma Questionnaire (for DSM-5); ICG = Inventory of Complicated Grief; ISI = Insomnia Severity Index; ITQ = International Trauma Questionnaire; K10 = Kessler Psychological Distress Scale; LASC = Los Angeles Symptom Checklist; MINI = Mini International Neuropsychiatric Interview; MLQ – Meaning in Life Questionnaire; MOS-20 = Medical Outcomes Study 20-Item; PCL/PCL-C/PCL-S/PCL-5 = PTSD Checklist (civilian version, stressor specific version; for DSM-5); PDS = Posttraumatic Diagnostic Scale; PG-13 = Prolonged Grief Disorder – 13 Scale; PHQ = Patient Health Questionnaire (2-/9-item scale for depression, 15-item scale for somatic symptoms; 4-item scale for depression and generalised anxiety); PRIME-MD = Primary Care Evaluation

of Mental Disorders; PSS-I/PSS-I-5 = PTSD Symptom Scale – Interview (for DSM-5); PSS-10 = Perceived Stress Scale; PTGI = Posttraumatic Growth Inventory; RAR-6 = Refugees' Anxiety Reasons – 6; RS-11 = Resilience Scale 11; SCID-P = Structured Clinical Interview for DSM-5 Disorders – PTSD Module; SF-12/SF-12v2 = Short-Form 12-Item Health Survey (version 2); SPTSS = Screen for Posttraumatic Stress Symptoms; SQD/SQD-D/SQD-P = Screening Questionnaire for Disaster Mental Health (depression and PTSD subscales); SRQ = Self Reporting Questionnaire; SUD = substance use disorder; TQ = Trauma Experience Questionnaire; TSSC = Traumatic Stress Symptom Checklist.

Supplement 4: Reference list of included studies

Abiama, E. E., Ifeagwazi, C. M., & Chukwuorji, J. C. (2022). Rates of occurrence and influence of trauma exposure on posttraumatic stress disorder symptoms among survivors of terrorist attacks in Northeast m. *International Journal of Mental Health and Addiction, 20*(4), 2478-2492. https://doi.org/10.1007/s11469-021-00527-w

Abukar, I. M., Asir Rage, A. A., & Warsame, M. O. (2025). Prevalence of Post-Traumatic Stress Disorder and Associated Factors Among Internally Displaced Persons (IDPS) In Mogadishu Cross-Sectional Study. *Psychology Research and Behavior Management*, 183-196. https://doi.org/10.2147/PRBM.S488388

Adhikari Baral, I. & K. C., B. (2019). Post traumatic stress disorder and coping strategies among adult survivors of earthquake, Nepal. *BMC Psychiatry, 19*, 118. https://doi.org/10.1186/s12888-019-2090-y

Aldabbour, B., Abuabada, A., Lahlouh, A. *et al.* Psychological impacts of the Gaza war on Palestinian young adults: a cross-sectional study of depression, anxiety, stress, and PTSD symptoms. *BMC Psychol* 12, 696 (2024). https://doi.org/10.1186/s40359-024-02188-5

Aldabbour B, El-Jamal M, Abuabada A, et al. The Psychological Toll of War and Forced Displacement in Gaza: A Study on Anxiety, PTSD, and Depression. *Chronic Stress*. 2025;9. https://doi.org/10.1177/24705470251334943

Al Ibraheem, B., Kira, I. A., Aljakoub, J., & Al Ibraheem, A. (2017). The health effect of the Syrian conflict on IDPs and refugees. *Peace and Conflict: Journal of Peace Psychology*, *23*(2), 140-152. https://doi.org/10.1037/pac0000247

Alejo, E. G., Rueda, G., Ortega, M., & Orozco, L. C. (2007). Estudio epidemiológico del trastorno por estrés postraumático en población desplazada por la violencia política en Colombia. *Universitas Psychologica, 6*(3), 623-636.

Ali, M., Farooq, N., Bhatti, M. A., & Kuroiwa, C. (2012). Assessment of prevalence and determinants of posttraumatic stress disorder in survivors of earthquake in Pakistan using Davidson Trauma Scale. *Journal of Affective Disorders*, *136*(3), 238-243. https://doi.org/10.1016/j.jad.2011.12.023

Ali, M., Mutavi, T., Mburu, J. M., & Mathai, M. (2023). Prevalence of posttraumatic stress disorder and depression among internally displaced persons in Mogadishu-Somalia. *Neuropsychiatric Disease and Treatment*, *19*,469-478. https://doi.org/10.2147/NDT.S398423

Aluh, D. O., Okoro, R. N., & Aigbogun, O. (2024). Correlates of substance use and dependence among internally displaced persons in Maiduguri, Nigeria. *Journal of Substance Use*, *29*(4), 610-616. https://doi.org/10.1080/14659891.2023.2213758

Amsalem, D., Haim-Nachum, S., Lazarov, A. *et al.* The effects of war-related experiences on mental health symptoms of individuals living in conflict zones: a longitudinal study. *Sci Rep* 15, 889 (2025). https://doi.org/10.1038/s41598-024-84410-3

Asnakew, S., Shumet, S., Ginbare, W., Legas, G., & Haile, K. (2019). Prevalence of post-traumatic stress disorder and associated factors among Koshe landslide survivors, Addis Ababa, Ethiopia: a community-based, cross-sectional study. *BMJ open*, *9*(6), e028550.

Awa, T. M., Ugbe, U. M. J., Onwusaka, O. C., Abua, E. E., & Esu, E. B. (2024). Correlates of post-traumatic stress disorder among adult residents of conflict-affected communities in Cross River State, Nigeria: a cross-sectional study. *BMJ Open*, *14*(3), e078851. https://doi.org/10.1136/bmjopen-2023-078851

Banal, R., Thappa, J., Shah, H. U., Hussain, A., Chowhan, A., Kaur, H., Bharti, M., & Thappa, S. (2010). Psychiatric morbidity in adult Kashmiri migrants living in a migrant camp at Jammu. *Indian Journal of Psychiatry*, *52*(2), 154-158. https://doi.org/10.4103/0019-5545.64597

Başoğlu, M., Şalcioğlu, E., & Livanou, M. (2002). Traumatic stress responses in earthquake survivors in Turkey. *Journal of Traumatic Stress*, *15*(4), 269-276. https://doi.org/10.1023/A:1016241826589

Bekeko, S. D., Nimani, T. D., Darcho, S. D., & Bayisa, F. S. (2025). Depression, anxiety and its predictor among internally displaced person in metekel Ethiopia, 2023: using a structural equation model. *Frontiers in Psychiatry*, *15*, 1458939. https://doi.org/10.3389/fpsyt.2024.1458939

Ben-Ezra, M., Goodwin, R., Leshem, E., & Hamama-Raz, Y. (2023). PTSD symptoms among civilians being displaced inside and outside the Ukraine during the 2022 Russian invasion. *Psychiatry Research*, *320*, 115011. https://doi.org/10.1016/j.psychres.2022.115011

Cerdá, M., Paczkowski, M., Galea, S., Nemethy, K., Péan, C., & Desvarieux, M. (2013). psychopathology in the aftermath of the h aiti earthquake: A population-based study of posttraumatic stress disorder and major depression. *Depression and Anxiety*, *30*(5), 413-424. https://doi.org/10.1002/da.22007

Cheung, A., Makhashvili, N., Javakhishvili, J., Karachevsky, A., Kharchenko, N., Shpiker, M., & Roberts, B. (2019). Patterns of somatic distress among internally displaced persons in Ukraine: analysis of a cross-sectional survey. *Social Psychiatry and Psychiatric Epidemiology*, *54*, 1265-1274. https://doi.org/10.1007/s00127-019-01652-7

Chukwuorji, J. C., Ifeagwazi, C. M., & Eze, J. E. (2017). Role of event centrality and emotion regulation in posttraumatic stress disorder symptoms among internally displaced persons. *Anxiety, Stress, & Coping*, *30*(6), 702-715. https://doi.org/10.1080/10615806.2017.1361936

Cofini, V., Carbonelli, A., Cecilia, M. R., Binkin, N., & di Orio, F. (2015). Post traumatic stress disorder and coping in a sample of adult survivors of the Italian earthquake. *Psychiatry Research*, *229*(1-2), 353-358. https://doi.org/10.1016/j.psychres.2015.06.041

Echenique, C., Medina, L. M., Medina, A. R., & Ramírez, A. (2008). Prevalencia del trastorno por estrés postraumático en población desplazada por violencia, en proceso de reestablecimiento en Sincelejo. *Psicología desde el Caribe*, *21*, 122-135.

Elhadi, M., Msherghi, A., Khaled, A., Alsoufi, A., Alhadi, A., Kareem, A., Ashini, A., Alsharif, T., Alhodiri, A., Altaeb, E., Hamed, M., Itrunbah, A., Mohmmed, S., Alameen, H., Idheiraj, H., Shuwayyah, A., Alhudhairy, S., Alansari, A., Abraheem, W., Akl, H., Nagib, T., Almugaddami, A.,

Aljameel, B., Muamr, S., Alsuwiyah, S., Alsghair, A., Soula, E., Buzreg, A., Alagelli, F., Aldireewi, A., Bareem, A., Alshareea, E., Gemberlo, A., & Zaid, A. (2022). Impact of lockdown due to the COVID-19 pandemic on mental health among the Libyan population. *PLoS One*, *17*(4), e0267426. https://doi.org/10.1371/journal.pone.0267426

Espinoza-Neyra, C., Jara-Pereda, M., & Díaz-Vélez, C. (2017). Post-traumatic stress disorder in a population affected by floods caused by el niño in Peru. *Revista Peruana de Medicina Experimental y Salud Publica*, *34*(4), 751-752. https://doi.org/10.17843/rpmesp.2017.344.3058

Farina, B., Mazzotti, E., Farina, F., Della Marca, G., Savoja, V., Kotzalidis, G. D., Campanile, A., Chemtob, C. M., Di Giannantonio, M., & Tatarelli, R. (2012). Relationship between handedness and persistent emotional distress in adults experiencing an earthquake. *Rivista di Psichiatria*, *47*(4), 309-312. https://doi.org/10.1708/1139.12558

Faronbi, J. O., Adegbola, G. A., Bello, C. B., Akinyoola, O. D., & Oginni, M. O. (2020). Posttraumatic stress disorder and suicidal ideation among the internally displaced persons in Nigeria. *Egyptian Nursing Journal*, *17*(3), 154-160. https://doi.org/10.4103/enj.enj_33_20

Freh, F.M., North, C.S. Posttraumatic stress disorder, general psychopathology, and suicidality among Yazidi women in Iraq kidnapped and/or raped by ISIS in 2014. *Arch Womens Ment Health* 28, 1221–1232 (2025). https://doi.org/10.1007/s00737-025-01594-x

Freitag, S., Strauß, K., Hannig, C., Rostalski, T., & Schmidt, S. (2011). Prädiktoren von Lebensqualität im Alter bei Menschen mit und ohne Vertreibungshintergrund im 2. Weltkrieg. *Zeitschrift für Medizinische Psychologie*, *20*(4), 170-177. https://doi.org/10.3233/zmp-2011-2031

Freitag, S., Braehler, E., Schmidt, S., & Glaesmer, H. (2013). The impact of forced displacement in World War II on mental health disorders and health-related quality of life in late life–a German population-based study. *International Psychogeriatrics*, *25*(2), 310-319. https://doi.org/10.1017/S1041610212001585

Garabiles, M. R., Mordeno, I. G., & Nalipay, M. J. N. (2023). A comparison of DSM-5 and ICD-11 models of PTSD: Measurement invariance and psychometric validation in Filipino trauma samples. *Journal of Psychiatric Research*, *163*, 24-31. https://doi.org/10.1016/j.jpsychires.2023.05.006

Gebreyesus, A., Gebremariam, A. G., Kidanu, K. G., Gidey, S., Haftu, H., Nigusse, A. T., Shishay, F., & Mamo, L. (2024). Post-traumatic stress disorder symptoms among internally displaced persons: unveiling the impact of the war of Tigray. *Discover Mental Health*, *4*(1), 18. https://doi.org/10.1007/s44192-024-00069-2

Gebreyesus, A., Niguse, A. T., Shishay, F., Mamo, L., Gebremedhin, T., Tsegay, K., Gebremariam, A. G., Kidanu, K.G., Gidey, S., & Tesfay, F. (2024). Prevalence of depression and associated factors among community hosted internally displaced people of Tigray; during war and siege. *BMC Psychiatry*, *24*(1), 3. https://doi.org/10.1186/s12888-023-05333-3

Ghafari-Nejad, A., Ahmadi-Mousavi, M., Gandomkar, M., & Reyhani-Kermani, H. (2007). The prevalence of complicated grief among Bam earthquake survivors in Iran. *Archives of Iranian Medicine*, *10*(4), 525-528.

Greene-Cramer, B., Summers, A., Lopes-Cardozo, B., Husain, F., Couture, A., & Bilukha, O. (2020). Noncommunicable disease burden among conflict-affected adults in Ukraine: a cross-sectional study of prevalence, risk factors, and effect of conflict on severity of disease and access to care. *PLoS One*, *15*(4), e0231899. https://doi.org/10.1371/journal.pone.0231899

Hagh-Shenas, H., Goodarzi, M. A., Farajpoor, M., & Zamyad, A. (2006). Post-traumatic stress disorder among survivors of Bam earthquake 40 days after the event. *Eastern Mediterranean Health Journal*, *12*(2), 118-125.

Hall, B. J., Xiong, Y. X., Yip, P. S., Lao, C. K., Shi, W., Sou, E. K., Chang, K., Wang, L., & Lam, A. I. (2019). The association between disaster exposure and media use on post-traumatic stress disorder following Typhoon Hato in Macao, China. *European Journal of Psychotraumatology*, *10*(1), 1558709. https://doi.org/10.1080/20008198.2018.1558709

Hamama, L., Sarid, O., & Hamama-Raz, Y. (2025). Psychological Distress, Resources, and Coping Strategies Among Evacuees and Non-Evacuees From an Armed Conflict Zone: A Network Analysis. *Stress and Health*, *41*(1), e3525. https://doi.org/10.1002/smi.3525

Hamid, A. A. & Musa, S. A. (2010). Mental health problems among internally displaced persons in Darfur. *International Journal of Psychology*, *45*(4), 278-285. https://doi.org/10.1080/00207591003692620

Hashmi, S., Petraro, P., Rizzo, T., Nawaz, H., Choudhary, R., Tessier-Sherman, B., Kasl, S., & Nawaz, H. (2011). Symptoms of anxiety, depression, and posttraumatic stress among survivors of the 2005 Pakistani earthquake. *Disaster Medicine and Public Health Preparedness*, *5*(4), 293-299. https://doi.org/10.1001/dmp.2011.81

Heeke, C., Stammel, N., & Knaevelsrud, C. (2015). When hope and grief intersect: Rates and risks of prolonged grief disorder among bereaved individuals and relatives of disappeared persons in Colombia. *Journal of Affective Disorders*, *173*, 59-64. https://doi.org/10.1016/j.jad.2014.10.038

Hemmeda, L., Ahmed, A.S., Fadlalmoula, G.A.G.A. *et al.* Displacement-related stressors in a Sudanese war-affected community; identifying the impact of war exposure and ongoing stressors on trauma symptom severity: a national multi-center cross-sectional study. *BMC Public Health* **25**, 1325 (2025). https://doi.org/10.1186/s12889-025-22411-1

Hodges, J. C., Maldonado-Molina, M. M., Schwartz, S. J., García, M. F., Pineros-Leano, M. F., Bates, M. M., Montero-Zamora, P., Calderón, I., Rodríguez, J., & Salas-Wright, C. P. (2025). The impact of hurricane trauma and cultural stress on posttraumatic stress among hurricane Maria survivors relocated to the U.S. mainland. *Cultural Diversity & Ethnic Minority Psychology, 31*(2), 233-244. https://doi.org/10.1037/cdp0000623

Howard, W. T., Loberiza, F. R., Pfohl, B. M., Thorne, P. S., Magpantay, R. L., & Woolson, R. F. (1999). Initial results, reliability, and validity of a mental health survey of Mount Pinatubo disaster victims. *The Journal of Nervous and Mental Disease*, *187*(11), 661-672. https://doi.org/10.1097/00005053-199911000-00003

Ibrahim, H., Ertl, V., Catani, C., Ismail, A. A., & Neuner, F. (2018). Trauma and perceived social rejection among Yazidi women and girls who survived enslavement and genocide. *BMC Medicine*, *16*, 154.https://doi.org/10.1186/s12916-018-1140-5

Ide-Okochi, A., Samiso, T., Kanamori, Y., He, M., Sakaguchi, M., & Fujimura, K. (2022). Depression, insomnia, and probable post-traumatic stress disorder among survivors of the 2016 Kumamoto earthquake and related factors during the recovery period amidst the COVID-19 pandemic. *International Journal of Environmental Research and Public Health*, *19*(7), 4403. https://doi.org/10.3390/ijerph19074403

Jann, P., Neldner, S., Neuner, F., & Mohammed, R. (2024). Complicated grief and posttraumatic stress after loss and separation under terror conditions. *Journal of TraumaticStress*, *37*(1), 154-165. https://doi.org/10.1002/jts.22990

Jayasuriya, D. (2014). Influence of posttraumatic growth on mental health and well-being across respondents severely affected by war in post-conflict Sri Lanka. *SocialIndicatorsResearch*, *119*, 265-280. https://doi.org/10.1007/s11205-013-0484-7

Johnson, R. J., Antonaccio, O., Botchkovar, E., & Hobfoll, S. E. (2022). War trauma and PTSD in Ukraine's civilian population: comparing urban-dwelling to internally displaced persons. *Social Psychiatry and Psychiatric Epidemiology*, *57*, 1807-1816. https://doi.org/10.1007/s00127-021-02176-9

Kakaje, A., Al Zohbi, R., Hosam Aldeen, O., Makki, L., Alyousbashi, A., & Alhaffar, M. B. A. (2021). Mental disorder and PTSD in Syria during wartime: a nationwide crisis. *BMC Psychiatry*, *21*, 2. https://doi.org/10.1186/s12888-020-03002-3

Karatzias, T., Shevlin, M., Ben-Ezra, M., McElroy, E., Redican, E., Vang, M. L., Cloitre, M., Ho, G. W. K., Lorberg, B., Martsenkovskyi, D., & Hyland, P. (2023). War exposure, posttraumatic stress disorder, and complex posttraumatic stress disorder among parents living in Ukraine during the Russian war. *Acta Psychiatrica Scandinavica*, *147*(3), 276-285. https://doi.org/10.1111/acps.13529

Khalil, K.A., Mohammed, G.T.F., Ahmed, A.B.M. *et al.* War-related trauma and posttraumatic stress disorder in refugees, displaced, and nondisplaced people during armed conflict in Sudan: a cross-sectional study. *Confl Health* 18, 66 (2024). https://doi.org/10.1186/s13031-024-00627-z

Kılıç, C., Aydın, İ., Taşkıntuna, N., Özçürümez, G., Kurt, G., Eren, E., Lale, T., Özel, S., & Zileli, L. (2006). Predictors of psychological distress in survivors of the 1999 earthquakes in Turkey: effects of relocation after the disaster. *Acta Psychiatrica Scandinavica*, *114*(3), 194-202. https://doi.org/10.1111/j.1600-0447.2006.00786.x

Kim, G., Torbay, R., & Lawry, L. (2007). Basic health, women's health, and mental health among internally displaced persons in Nyala Province, South Darfur, Sudan. *American Journal of Public Health*, *97*(2), 353-361. https://doi.org/10.2105/AJPH.2005.073635

King, R. V., Polatin, P. B., Hogan, D., Downs, D. L., & North, C. S. (2016). Needs assessment of Hurricane Katrina evacuees residing temporarily in Dallas. *Community MentalHealthJournal*, *52*, 18-24. https://doi.org/10.1007/s10597-015-9938-5

Kira, I., Aljakoub, J., Al Ibraheem, B., & Shuwiekh, H. (2023). The effects of type III traumatic stressors of the protracted conflict and prolonged COVID-19 on Syrians internally displaced: A validation study of type III continuous traumatic stressors and their impact. *International Perspectives in Psychology: Research, Practice, Consultation, 12*(1), 1-15. https://doi.org/10.1027/2157-3891/a000054

Kozarić-Kovacić, D., Ljubin, T., & Grappe, M. (2000). Comorbidity of posttraumatic stress disorder and alcohol dependence in displaced persons. *Croatian Medical Journal*, *41*(2), 173-178.

Kumar, M. S., Murhekar, M. V., Hutin, Y., Subramanian, T., Ramachandran, V., & Gupte, M. D. (2007). Prevalence of posttraumatic stress disorder in a coastal fishing village in Tamil Nadu, India, after the December 2004 tsunami. *AmericanJournal of PublicHealth*, *97*(1), 99-101. https://doi.org/10.2105/AJPH.2005.071167

Kun, P., Chen, X., Han, S., Gong, X., Chen, M., Zhang, W., & Yao, L. (2009). Prevalence of post-traumatic stress disorder in Sichuan Province, China after the 2008 Wenchuan earthquake. *Public Health*, *123*(11), 703-707. https://doi.org/10.1016/j.puhe.2009.09.017

Kuo, H. W., Wu, S. J., Ma, T. C., Chiu, M. C., & Chou, S. Y. (2007). Posttraumatic symptoms were worst among quake victims with injuries following the Chi-chi quake in Taiwan. *Journal of Psychosomatic Research*, *62*(4), 495-500. https://doi.org/10.1016/j.jpsychores.2004.11.012

Kuwert, P., Brähler, E., Glaesmer, H., Freyberger, H. J., & Decker, O. (2009). Impact of forced displacement during World War II on the present-day mental health of the elderly: a population-based study. *International Psychogeriatrics*, *21*(4), 748-753. https://doi.org/10.1017/S1041610209009107

Larrance, R., Anastario, M., & Lawry, L. (2007). Health status among internally displaced persons in Louisiana and Mississippi travel trailer parks. *Annals of EmergencyMedicine*, *49*(5), 590-601. https://doi.org/10.1016/j.annemergmed.2006.12.004

Lê, F., Tracy, M., Norris, F. H., & Galea, S. (2013). Displacement, county social cohesion, and depression after a large-scale traumatic event. *SocialPsychiatry and PsychiatricEpidemiology*, *48*, 1729-1741. https://doi.org/10.1007/s00127-013-0698-7

Liu, X., Ma, X., Hu, X., Qiu, C., Wang, Y., Wang, Q., Zhang, W., Zhang, J., & Li, T. (2012). A risk score for predicting post-traumatic stress disorder in adults in a Chinese earthquake area. *Journal of International Medical Research*, *40*(6), 2191-2198. https://doi.org/10.1177/030006051204000617

Lopes Cardozo B, Vergara A, Agani F, Gotway CA. Mental health, social functioning, and attitudes of Kosovar Albanians following the war in Kosovo. JAMA. 2000 Aug 2;284(5):569-77. doi: 10.1001/jama.284.5.569

Lushchak, O., Velykodna, M., Bolman, S., Strilbytska, O., Berezovskyi, V., & Storey, K. B. (2024). Prevalence of stress, anxiety, and symptoms of post-traumatic stress disorder among Ukrainians after the first year of Russian invasion: a nationwide cross-sectional study. *The Lancet Regional Health – Europe*, *36*, 100773. https://doi.org/10.1016/j.lanepe.2023.100773

Ma, N., Xiang, H., Wang, R., He, M., Cheng, Z., Cui, L., Liang, G., Yang, L., Liu, Y., Yan, B., Zhang, Y., Li, L., Ma, H., & He, Y. (2010). Reliability and validity of the Chinese version of the Mini-International Neuropsychiatric Interview for screening posttraumatic stress disorder among earthquake survivors. *Chinese Mental Health Journal*, *24*(9), 643-646. https://doi.org/10.3969/j.issn.1000-6729.2010.09.001

Ma, N., Ma, H., He, H., Yu, X., & Caine, E. D. (2013). Characteristics of Wenchuan earthquake victims who remained in a government-supported transitional community. *Asia-Pacific Psychiatry*, *5*(2), E73-E80. https://doi.org/10.1111/appy.12074

Madoro, D., Kerebih, H., Habtamu, Y., G/tsadik, M., Mokona, H., Molla, A., Wondie, T., & Yohannes, K. (2020). Post-traumatic stress disorder and associated factors among internally displaced people in South Ethiopia: a cross-sectional study. *Neuropsychiatric Disease and Treatment*, *16*, 2317-2326. https://doi.org/10.2147/NDT.S267307

Makango, B., Alemu, Z. A., Solomon, T., Lemma, N., Girma, T., Mohammednur, T., Alayu, M., & Fufa, Y. (2023). Prevalence and factors associated with post-traumatic stress disorder among internally displaced people in camps at Debre Berhan, Amhara Region, Ethiopia: a cross-sectional study. *BMC Psychiatry*, *23*, 81. https://doi.org/10.1186/s12888-023-04570-w

Makhashvili, N., Chikovani, I., McKee, M., Bisson, J., Patel, V., & Roberts, B. (2014). Mental disorders and their association with disability among internally displaced persons and returnees in Georgia. *Journal of Traumatic Stress*, *27*(5), 509-518. https://doi.org/10.1002/jts.21949

Mamed GE, Tefera GM, Bitew M, Yu M. The overlooked war in Northern Ethiopia: Examining psychological capital, mental distress, and post-traumatic stress disorder among internally displaced people in Amhara region. *International Journal of Social Psychiatry*. 2024;71(4):705-714. doi:10.1177/00207640241299357

Manafe, N., Ismael-Mulungo, H., Ponda, F., Dos Santos, P. F., Mandlate, F., Cumbe, V. F., Mocumbi, A. O., & Oliveira Martins, M. R. (2024). Prevalence and associated factors of common mental disorders among internally displaced people by armed conflict in Cabo Delgado, Mozambique: a cross-sectional community-based study. *Frontiers in Public Health*, *12*, 1371598. https://doi.org/10.3389/fpubh.2024.1371598

Matsuoka, Y., Haseda, M., Kanamori, M., Sato, K., Amemiya, A., Ojima, T., Takagi, D., Hazanato, M., & Kondo, N. (2023). Does disaster-related relocation impact mental health via changes in group participation among older adults? Causal mediation analysis of a pre-post disaster study of the 2016 Kumamoto earthquake. *BMCPublicHealth*, *23*,1982. https://doi.org/10.1186/s12889-023-16877-0

Matthews, V., Longman, J., Berry, H. L., Passey, M., Bennett-Levy, J., Morgan, G. G., Pit, S., Rolfe, M., & Bailie, R. S. (2019). Differential mental health impact six months after extensive river flooding in rural Australia: a cross-sectional analysis through an equity lens. *Frontiers in Public Health*, *7*, 367. https://doi.org/10.3389/fpubh.2019.00367

McGinty, G., Fox, R., Roberts, B., Makhashvili, N., Javakhishvili, J. D., & Hyland, P. (2023). Post-traumatic stress disorder, complex post-traumatic stress disorder, and coping styles among internally

displaced Ukrainians. *Journal of Loss and Trauma*, *28*(7), 571-587. https://doi.org/10.1080/15325024.2023.2217002

Melese, M., Maru, L., & Esubalew, D. (2024). Posttraumatic stress disorder and its associated factors among people living in Dabat district, northwest Ethiopia. *Frontiers in Psychiatry*, *15*, 1359382. https://doi.org/10.3389/fpsyt.2024.1359382

Melkam, M., Medfu Takelle, G., Kibralew, G., & Nakie, G. (2025). Post-traumatic stress disorder and its associated factors among internally displaced people due to conflict in Northwest Ethiopia. *Frontiers in Public Health*, *13*, 1386566. https://doi.org/10.3389/fpubh.2025.1386566

Mirkhan Ahmed, H., Ahmed Abdulla, S., Al-Tawil, N., & Mishkin, K. (2024). Maternal health experiences, depression, and anxiety among refugees and displaced persons in Iraq: a cross-sectional study. *Cureus*, *16*(8), e67645. https://doi.org/10.7759/cureus.67645

Mohamed, E. H. & Kheir, D. A. (2024). Prevalence of post-traumatic stress disorder and depression and associated factors among internally displaced persons in Al-Galgala, Sudan. *Neuropsychiatric Disease and Treatment*, *20*,1155-1168. https://doi.org/10.2147/NDT.S462342

Monsalve, S. D., Vargas-Monroy, A. M., Ariza, J. E., Oñate Cuello, A. M., Ropero Vera, A. R., Bermudez Cuello, J. C., Zuleta, L. A., Novella, A. F. C., Quintero, E. P., Ortiz, Y. N. F., Carillo, M. A., & Kroeger, A. (2022). Mental health among displaced and non-displaced populations in Valledupar, Colombia: do inequalities continue? *Pathogens and GlobalHealth*, *116*(5), 305-318. https://doi.org/10.1080/20477724.2021.1989186

Montero-Zamora, P., Salas-Wright, C. P., Maldonado-Molina, M. M., Brown, E. C., Vos, S. R., Garcia, M. F., Scaramutti, C., José, R., Bates, M. M., & Schwartz, S. J. (2023). Hurricane stress, cultural stress, and mental health among hurricane Maria migrants in the US mainland. *American Journal of Orthopsychiatry*, *93*(3), 211-224. https://doi.org/10.1037/ort0000669

Muhtz, C., von Alm, C., Godemann, K., Wittekind, C., Jelinek, L., Yassouridis, A., & Kellner, M. (2011). Long-term consequences of flight and expulsion in former refugee children. *Psychotherapie, Psychosomatik, Medizinische Psychologie*, *61*(5), 233-238. https://doi.org/10.1055/s-0031-1275341

Muhummed, A. M., Jibril, M. K., Yimam, A. A., & Ali, S. Y. (2024). Prevalence and correlates of post-traumatic stress disorder among internal displaced people in Qoloji Camps, Somali regional state, Eastern Ethiopia. *The International Journal of Psychiatry in Medicine*, *60*(1), 17-32. https://doi.org/10.1177/00912174241232001

Mumin, F. I., Wesonga, F. D., Handuleh, J. I., White, R. G., & Mor, S. M. (2022). COVID-19 and its prevention in internally displaced person (IDP) camps in Somalia: impact on livelihood, food security and mental health. *BMC Public Health*, *22*, 2414. https://doi.org/10.1186/s12889-022-14878-z

Naeem, F., Ayub, M., Masood, K., Gul, H., Khalid, M., Farrukh, A., Shaheen, A., Waheed, W., & Chaudhry, H. R. (2011). Prevalence and psychosocial risk factors of PTSD: 18 months after Kashmir earthquake in Pakistan. *Journal of Affective Disorders*, *130*(1-2), 268-274. https://doi.org/10.1016/j.jad.2010.10.035

Pabón-Poches, D. K., Barchelot-Aceros, L., Galván, G., De la Hoz, F. V., Guerrero-Martelo, M. F., & Navarro, L. V. S. (2024). La Polivictimización, la Presencia de Síntomas TEPT y la Afectación en la Funcionalidad de las Personas que Son Víctimas del Desplazamiento Forzado: Estudio Comparativo. *Anuario de Psicología Jurídica*, *34*(1), 13-22. https://doi.org/10.5093/apj2024a4

Peleg, O., & Gendelman, L. (2025). Early Evidence on the Emotional Distress of Civilians, Including Evacuees, During a Recent Conflict. *International Journal of Psychology*, *60*(3), e70048. https://doi.org/10.1002/ijop.70048

Pinchuk, I., Solonskyi, A., Yachnik, Y., Kopchak, O., Klasa, K., Sobański, J. A., & Odintsova, T. (2024). Psychological well-being of Ukrainian students three months after the emerge of full-scale war. *Psychiatria Polska*, *58*(1), 121-151. https://doi.org/10.12740/PP/177073

Polyvianaia, M., Yachnik, Y., Fegert, J.M. *et al.* Mental health of university students twenty months after the beginning of the full-scale Russian-Ukrainian war. *BMC Psychiatry* 25, 236 (2025). https://doi.org/10.1186/s12888-025-06654-1

Rajkumari, B., Ingudam, D., Yengkokpam, C., Oinam, G., & Yumnam, R. (2024). Psychological effects of Manipur violence among the internally displaced persons residing in relief camps across Imphal valley of Manipur–A cross-sectional study. *Journal of Family Medicine and Primary Care*, *13*(10), 4354-4362. DOI: 10.4103/jfmpc.jfmpc_341_24

Ramachandran, A., Makhashvili, N., Javakhishvili, J., Karachevskyy, A., Kharchenko, N., Shpiker, M., Ezard, N., Fuhr, D. C., & Roberts, B. (2019). Alcohol use among conflict-affected persons in Ukraine: risk factors, coping and access to mental health services. *EuropeanJournal of PublicHealth*, *29*(6), 1141-1146. https://doi.org/10.1093/eurpub/ckz117

Rana, H., Ali, S., Yusufi, B., Alexander, D. A., Klein, S., Lee, A. J., Jones, G. T., & Macfarlane, G. (2008). The psychological and psychosocial impact of the Pakistan Kashmir earthquake after 8 months: a preliminary evaluation by PACTT: PACTT: Pakistan—Aberdeen Collaborative Trauma Team. *International Psychiatry*, *5*(2), 43-46. https://doi.org/10.1192/S1749367600005609

Ranasinghe, P. D. & Levy, B. R. (2007). Prevalence of and sex disparities in posttraumatic stress disorder in an internally displaced Sri Lankan population 6 months after the 2004 Tsunami. *Disaster Medicine and Public Health Preparedness*, *1*(1), 34-41. https://doi.org/10.1097/DMP.0b013e318068fbb7

Richa, S., Herdane, M., Dwaf, A., Bou Khalil, R., Haddad, F., El Khoury, R., Zarzour, M., Kassab, A., Dagher, R., Brunet, A., & El-Hage, W. (2020). Trauma exposure and PTSD prevalence among Yazidi, Christian and Muslim asylum seekers and refugees displaced to Iraqi Kurdistan. *PLoS One*, *15*(6), e0233681. https://doi.org/10.1371/journal.pone.0233681

Richards, A., Ospina-Duque, J., Barrera-Valencia, M., Escobar-Rincón, J., Ardila-Gutiérrez, M., Metzler, T., & Marmar, C. (2011). Posttraumatic stress disorder, anxiety and depression symptoms, and psychosocial treatment needs in Colombians internally displaced by armed conflict: A mixed-method evaluation. *Psychological Trauma: Theory, Research, Practice, and Policy*, *3*(4), 384-393.https://doi.org/10.1037/a0022257

Roberts, B., Ocaka, K. F., Browne, J., Oyok, T., & Sondorp, E. (2008). Factors associated with post-traumatic stress disorder and depression amongst internally displaced persons in northern Uganda. *BMC Psychiatry*, *8*, 38. https://doi.org/10.1186/1471-244X-8-38

Roberts, B., Ocaka, K. F., Browne, J., Oyok, T., & Sondorp, E. (2011). Alcohol disorder amongst forcibly displaced persons in northern Uganda. *AddictiveBehaviors*, *36*(8), 870-873. https://doi.org/10.1016/j.addbeh.2011.03.006

Robinson, M., McGlinchey, E., Ardila, Y., Guillen, F., Acosta, N., Gomez, J., ... & Armour, C. (2025). Estudio De La Vida Bajo Estres: methodological overview and baseline data analysis of a case-control investigation of risk and resiliency factors for traumatic stress in Colombia. *Journal of Psychopathology and Behavioral Assessment*, *47*(1), 25. https://doi.org/10.1007/s10862-025-10203-1

Roitblat, Y., Nehuliaieva, L., Nedilko, R., Shashkov, Y., & Shterenshis, M. (2024). Immediate mental health status of refugees during the Ukrainian armed conflict of 2022. *American Journal of Orthopsychiatry*, *95*(2), 115-125. https://doi.org/10.1037/ort0000768

Salah, T. T. M., Abdelrahman, A., Lien, L., Eide, A. H., Martinez, P., & Hauff, E. (2013). The mental health of internally displaced persons: an epidemiological study of adults in two settlements in Central Sudan. *International Journal of Social Psychiatry*, *59*(8), 782-788. https://doi.org/10.1177/0020764012456810

Şalcıoğlu, E., Başoğlu, M., & Livanou, M. (2003). Long-term psychological outcome for non-treatment-seeking earthquake survivors in Turkey. *The Journal of Nervous and Mental Disease*, *191*(3), 154-160. https://doi.org/10.1097/01.NMD.0000054931.12291.50

Şalcıoğlu, E., Başoğlu, M., & Livanou, M. (2007). Post-traumatic stress disorder and comorbid depression among survivors of the 1999 earthquake in Turkey. *Disasters*, *31*(2), 115-129. https://doi.org/10.1111/j.1467-7717.2007.01000.x

Şalcıoğlu, E., Ozden, S., & Ari, F. (2018). The role of relocation patterns and psychosocial stressors in posttraumatic stress disorder and depression among earthquake survivors. *The Journal of Nervous and Mental Disease*, *206*(1), 19-26. https://doi.org/10.1097/NMD.0000000000000627

Scaramutti, C., Salas-Wright, C. P., Vos, S. R., & Schwartz, S. J. (2019). The mental health impact of hurricane Maria on Puerto Ricans in Puerto Rico and Florida. *Disaster Medicine and PublicHealthPreparedness*, *13*(1), 24-27. https://doi.org/10.1017/dmp.2018.151

Shehab, N., Anastario, M. P., & Lawry, L. (2008). Access To Care Among Displaced Mississippi Residents In FEMA Travel Trailer Parks Two Years After Katrina. *Health Affairs*, *27*(5), w416-w429. https://doi.org/10.1377/hlthaff.27.5.w416

Shevchenko, R., Kaafarani, A., Aliieva, T., Piletska, L., Radul, I., Klepar, M., & Popovych, I. (2020). Features of the psychoemotional state of internally displaced families from the war zone. *Revista Inclusiones*, *7*,157-169.

Shiga, T., Zhang, W., Ohira, T., Suzuki, Y., Maeda, M., Mashiko, H., Yabe, H., Iwasa, H., Nakano, H., Yasumura, S., Kamiya, K., & Fukushima Health Management Survey Group. (2021).

Socioeconomic status, damage-related conditions, and PTSD following the Fukushima-daiichi nuclear power plant accident: The Fukushima Health Management Survey. *Fukushima Journal of Medical Science*, *67*(2), 71-82. https://doi.org/10.5387/fms.2020-24

Sinisterra Mosquera, M., Figueroa Lozano, F. S., Moreno Gutiérrez, V. F., Robayo, M. F., & Sanguino Leal, J. F. (2010). Prevalencia del trastorno de estrés post traumático en población en situación de desplazamiento en la localidad de Ciudad Bolívar Bogotá, Colombia 2007. *Psychologia. Avances de la Disciplina*, *4*(2), 83-97. https://doi.org/10.21500/19002386.1147

Siriwardhana, C., Adikari, A., Pannala, G., Roberts, B., Siribaddana, S., Abas, M., Sumathipala, A., & Stewart, R. (2015). Changes in mental disorder prevalence among conflict-affected populations: a prospective study in Sri Lanka (COMRAID-R). *BMC Psychiatry*, *15*, 41. https://doi.org/10.1186/s12888-015-0424-y

Spell, A. W., Kelley, M. L., Wang, J., Self-Brown, S., Davidson, K. L., Pellegrin, A.,Palcic, J. L.,Meyer, K., Paasch, V., & Baumeister, A. (2008). The moderating effects of maternal psychopathology on children's adjustment post–Hurricane Katrina. *Journal of Clinical Child & Adolescent Psychology*, *37*(3), 553-563. https://doi.org/10.1080/15374410802148210

Sprang, G. & LaJoie, A. S. (2009). Exposure, avoidance, and PTSD among Hurricane Katrina evacuees. *Traumatology*, *15*(2), 10-19. https://doi.org/10.1177/1534765609331607

Strauss, K., Dapp, U., Anders, J., von Renteln-Kruse, W., & Schmidt, S. (2011). Range and specificity of war-related trauma to posttraumatic stress; depression and general health perception: displaced former World War II children in late life. *Journal of Affective Disorders*, *128*(3), 267-276. https://doi.org/10.1016/j.jad.2010.07.009

Suzuki, T., Akaishi, T., Nemoto, H., Utsumi, Y., Seto, M., Usukura, H., Kunii, Y., Sugawara, Y., Nakaya, N., Nakamura, T., Tsuchiya, N., Narita, A., Kogure, M., Hozawa, A., Tsuji, I., Ishii, T., & Tomita, H. (2021). Impact of type of reconstructed residence on social participation and mental health of population displaced by disasters. *ScientificReports*, *11*, 21465. https://doi.org/10.1038/s41598-021-00913-3

Tadesse, G., Gashaw, F., Zeleke, T. A., Fentahun, S., & Yitayih, S. (2024). Prevalence and factors associated with suicidal ideation and attempts among war-affected internally displaced people in northwest Ethiopia, 2022. *BJPsychOpen*, *10*(4), e132. https://doi.org/10.1192/bjo.2024.71

Taha, P. H. & Sijbrandij, M. (2021). Gender differences in traumatic experiences, PTSD, and relevant symptoms among the Iraqi internally displaced persons. *InternationalJournal of EnvironmentalResearch and PublicHealth*, *18*(18), 9779. https://doi.org/10.3390/ijerph18189779

Takahashi, S., Nakamura, M., Yonekura, Y., Tanno, K., Sakata, K., Ogawa, A., & Kobayashi, S. (2016). Association between relocation and changes in cardiometabolic risk factors: a longitudinal study in tsunami survivors of the 2011 Great East Japan Earthquake. *BMJ Open*, *6*(5), e011291. https://doi.org/10.1136/bmjopen-2016-011291

Tekeli-Yesil, S., Isik, E., Unal, Y., Aljomaa Almossa, F., Konsuk Unlu, H., & Aker, A. T. (2018). Determinants of mental disorders in Syrian refugees in Turkey versus internally displaced persons in

Syria. *American Journal of Public Health*, *108*(7), 938-945. https://doi.org/10.2105/AJPH.2018.304405

Thapa, S. B. & Hauff, E. (2005). Psychological distress among displaced persons during an armed conflict in Nepal. *Social Psychiatry and Psychiatric Epidemiology*, *40*, 672-679. https://doi.org/10.1007/s00127-005-0943-9

Timmer, A., Johnson, R. J., Antonaccio, O., Botchkovar, E. V., & Hughes, L. A. (2023). Forced to flee out and down: Depression following the Russian invasion and displacement of the Ukrainian people. *Peace and Conflict: Journal of Peace Psychology*, *29*(3), 266-275. https://doi.org/10.1037/pac0000689

Tiyuri, A., Rasoulian, M., Hajebi, A., Naserbakht, M., Shabani, A., Hakim Shooshtari, M., Rezapour, A., & Motevalian, S. A. (2023). Psychological impact of the Spring 2019 flood among adult population of Iran. *International Journal of Social Psychiatry*, *69*(8), 1916-1927. https://doi.org/10.1177/00207640231180824

Tsuchiya, M., Aida, J., Watanabe, T., Shinoda, M., Sugawara, Y., Tomata, Y., Yabe, Y., Sekiguchi, T., Watanabe, M., Osaka, K., Sasaki, K., Hagiwara, Y., & Tsuji, I. (2019). High prevalence of toothache among Great East Japan Earthquake survivors living in temporary housing. *Community Dentistry and Oral Epidemiology*, *47*(2), 119-126. https://doi.org/10.1111/cdoe.12433

Tural, Ü., Coşkun, B., Önder, E., Çorapçioğlu, A., Yildiz, M., Kesepara, C., Karakaya, I., Aydin, M., Erol, A., Torun, F., & Aybar, G. (2004). Psychological consequences of the 1999 earthquake in Turkey. *Journal of Traumatic Stress*, *17*(6), 451-459. https://doi.org/10.1007/s10960-004-5793-9

Valenti, M., Fujii, S., Kato, H., Masedu, F., Tiberti, S., & Sconci, V. (2013). Validation of the Italian version of the Screening Questionnaire for Disaster Mental Health (SQD) in a post-earthquake urban environment. *Annali dell'Istituto Superiore di Sanità*, *49*, 79-85. https://doi.org/10.4415/ANN_13_01_13

van Griensven, F., Chakkraband, M. S., Thienkrua, W., Pengjuntr, W., Cardozo, B. L., Tantipiwatanaskul, P., Mock, P. A., Ekassawin, S., Varangrat, A., Gotway, C., Sabin, M., Tappero, J. W., & Thailand Post-Tsunami Mental Health Study Group. (2006). Mental health problems among adults in tsunami-affected areas in southern Thailand. *Jama*, *296*(5), 537-548. https://doi.org/10.1001/jama.296.5.537

Wang, L., Zhang, Y., Wang, W., Shi, Z., Shen, J., Li, M., & Xin, Y. (2009). Symptoms of posttraumatic stress disorder among adult survivors three months after the Sichuan earthquake in China. *Journal of Traumatic Stress*, *22*(5), 444-450. https://doi.org/10.1002/jts.20439

Wani, S. M., Khan, A. W., Suhaff, A. A., & Guroo, M. A. (2020). Prevelance of PTSD in people affected by flood in Kashmir. *International Journal of Advances in Medical Sciences*, *4*(5), 1-6.

Wen, J., Shi, Y. K., Li, Y. P., Yuan, P., & Wang, F. (2012). Quality of life, physical diseases, and psychological impairment among survivors 3 years after Wenchuan earthquake: a population based survey. *PLoS One*, *7*(8), e43081. https://doi.org/10.1371/journal.pone.0043081

Yilmaz, E., Erdem, M. Post-traumatic stress disorder in earthquake survivors living in temporary shelter areas in Hatay central districts: a cross-sectional study. *BMC Psychiatry* 25, 461 (2025). https://doi.org/10.1186/s12888-025-06919-9

Zasiekina, L., Zasiekin, S., & Kuperman, V. (2023). Post-traumatic stress disorder and moral injury among Ukrainian civilians during the ongoing war. *Journal of CommunityHealth*, *48*, 784-792. https://doi.org/10.1007/s10900-023-01225-5

Zhang, Z., Shi, Z., Wang, L., & Liu, M. (2011). One year later: Mental health problems among survivors in hard-hit areas of the Wenchuan earthquake. *PublicHealth*, *125*(5), 293-300. https://doi.org/10.1016/j.puhe.2010.12.008

Zhang, Z., Wang, W., Shi, Z., Wang, L., & Zhang, J. (2012). Mental health problems among the survivors in the hard-hit areas of the Yushu earthquake. *PLoS One*, *7*(10), e46449. https://doi.org/10.1371/journal.pone.0046449

Zrineh A, Karmi H, Zeidan S, Salameh A, Ikhmayyes I. Interplay of post-traumatic stress disorder, post-traumatic growth, and social support among Palestinian refugees in West Bank camps: A cross-sectional study. *International Journal of Social Psychiatry*. 2025;0(0). doi:10.1177/00207640251341594

Supplement 5: List of excluded studies with reasons for exclusion

Study	Title	Reason for Exclusion
**Abbara et al., 2016**	Mental Health among displaced Syrians: findings from the Syria Public Health Network	No primary research
**Abello-Llanos et al., 2009**	Well-Being and Trauma in Adults Displaced by Political Violence	No primary research
**Acierno et al., 2006**	Risk and protective factors for psychopathology among older versus younger adults after the 2004 Florida hurricanes	Not focusing on point prevalences
**Acierno et al., 2007**	Psychological sequelae resulting from the 2004 Florida hurricanes: implications for postdisaster intervention.	N < 100
**Acosta et al., 2019**	Mental Health of Adolescents and Young People Victims of Forced Displacement in Colombia	not focusing on point prevalences
**Adams et al., 2011**	Psychological well-being and risk perceptions of mothers in Kyiv, Ukraine, 19 years after the Chornobyl disaster	not focusing on point prevalences
**Adeola, Fo et al., 2009**	Mental Health & Psychosocial Distress of Katrina: An Empirical Study of Survivors	no mental disorder according to ICD/DSM
**Adonis et al., 2018**	Acute stress disorder in Greek Cypriots visiting the occupied areas	N < 100
**Agani et al., 2010**	Preoccupation with Suicidal Thoughts in the Population of Kosovo 15 Years of Age and Older	no data on internally displaced individuals
**Agrawal et al., 2015**	Associations Between Sociodemographic Characteristics, Pre Migratory and Migratory Factors and Psychological Distress Just After Migration and After Resettlement: The Indian Migration Study.	no data on internally displaced individuals
**Agyapong et al., 2021**	Prevalence rates and correlates of likely post-traumatic stress disorder in residents of Fort McMurray 6 months after a wildfire	no data on internally displaced individuals
**Ahearn et al., 2004**	Post-civil war adaptation and need in Managua, Nicaragua.	no validated instrument based on DSM/ICD
**Ahmad et al., 2000**	Posttraumatic stress disorder in children after the military operation "Anfal" in Iraqi Kurdistan.	N < 100
**Akaishi et al., 2022**	Five-Year Psychosocial Impact of Living in Postdisaster Prefabricated Temporary Housing.	no useful comparison data
**Akhunzada et al., 2012**	Determinants of Post Traumatic Stress Disorder in Adults among Internally Displaced Persons of Bajaur and Swat	no peer-reviewed journal article
**Alberto et al., 2021**	Prevalence and factors associated with post-traumatic stress disorder in displaced persons in Colombia	not focusing on point prevalences
**Ali et al., 2013**	Mental health and the civil conflicts in Sudan.	no primary research
**Aljundi, 2018**	Mental health of Syrians: Internally displaced and refugees seven years of continuous suffering	no primary research
**Alsamara et al., 2024**	Exploring Women's Religious Coping Strategies and Psychological Impact After the 2023 Earthquake in Syria.	not focusing on point prevalences

**Amone-P'Olak et al., 2018**	PTSD, Mental Illness, and Care Among Survivors of Sexual Violence in Northern Uganda: Findings From the WAYS Study	no validated instrument based on DSM/ICD
**An et al., 2018**	Prevalence and Correlates of Suicidal Thoughts and Behaviors among North Korean Defectors.	no data on internally displaced individuals
**Anastario et al., 2009**	Increased gender-based violence among women internally displaced in Mississippi 2 years post-Hurricane Katrina.	not focusing on point prevalences
**Anastario et al., 2008**	Using mental health indicators to identify postdisaster gender-based violence among women displaced by Hurricane Katrina.	not focusing on point prevalences
**Anbesaw et al., 2024**	Factors associated with depression among war-affected population in Northeast, Ethiopia	N < 100
**Ayazi et al., 2014**	Association between exposure to traumatic events and anxiety disorders in a post-conflict setting: a cross-sectional community study in South Sudan	sample duplicate
**Ayazi et al., 2012**	What are the risk factors for the comorbidity of posttraumatic stress disorder and depression in a war-affected population? a cross-sectional community study in South Sudan	no data on internally displaced individuals
**Badri et al., 2012**	Exposures to war-related traumatic events and post-traumatic stress disorder symptoms among displaced Darfuri female university students: an exploratory study.	N < 100
**Bailie et al., 2022**	Exposure to risk and experiences of river flooding for people with disability and carers in rural Australia: a cross-sectional survey.	no data on internally displaced individuals
**Bamrah et al., 2013**	The impact of post-election violence on HIV and other clinical services and on mental health-Kenya, 2008.	no validated instrument based on DSM/ICD
**Başoğlu et al., 2005**	Psychiatric and Cognitive Effects of War in Former Yugoslavia: Association of Lack of Redress for Trauma and Posttraumatic Stress Reactions	no useful comparison data
**Belleville et al., 2021**	Psychological Symptoms Among Evacuees From the 2016 Fort McMurray Wildfires: A Population-Based Survey One Year Later.	no data on internally displaced individuals
**Belleville et al., 2019**	Post-Traumatic Stress among Evacuees from the 2016 Fort McMurray Wildfires: Exploration of Psychological and Sleep Symptoms Three Months after the Evacuation.	no data on internally displaced individuals
**Ben-Tzur et al., 2021**	Mastery, Social Support, and Sense of Community as Protective Resources Against Psychological Distress Among Israelis Exposed to Prolonged Rocket Attacks.	no data on internally displaced individuals
**Beshr et al., 2024**	The prevalence of depression and anxiety among medical students in Yemen: A cross-sectional study	no data on internally displaced individuals
**Besser et al., 2010**	The effects of insecure attachment orientations and perceived social support on posttraumatic stress and depressive symptoms among civilians exposed to the 2009 Israel-Gaza war: A follow-up cross-lagged panel design study	no data on internally displaced individuals
**Bland et al., 1996**	Long-term psychological effects of natural disasters.	no validated instrument based on DSM/ICD
**Bland et al., 2005**	Long-term follow-up of psychological distress following earthquake experiences among working Italian males: a cross-sectional analysis.	no data on internally displaced individuals
**Boiko et al., 2024**	Mental health and sleep disturbances among Ukrainian refugees in the context of Russian-Ukrainian war: A preliminary result from online-survey.	no data on internally displaced individuals
**Bonilla-Escobar et al., 2021**	Impacts of violence on the mental health of Afro-descendant survivors in Colombia.	sample not representative

**Bowler et al., 2017**	Posttraumatic stress disorder, gender, and risk factors: World Trade Center tower survivors 10 to 11 years after the September 11, 2001 attacks	no data on internally displaced individuals
**Boztas et al., 2019**	Effects of blast exposure on anxiety and symptoms of post-traumatic stress disorder (PTSD) among displaced Ukrainian populations.	no validated instrument based on DSM/ICD
**Bromet et al., 2011**	Growing up in the shadow of Chornobyl: adolescents' risk perceptions and mental health.	no data on internally displaced individuals
**Brown et al., 2023**	Beliefs About Traumatic Memories, Thought Control Strategies, and the Impact on PTSD Symptoms After a Natural Disaster.	not focusing on point prevalences
**Bryce et al., 1989**	Life experiences, response styles and mental health among mothers and children in Beirut, Lebanon.	not focusing on point prevalences
**Cabas-Hoyos et al., 2016**	Prevalence of post-traumatic stress disorder and associated events in adults victim of displacement in the Colombian Caribbean	no peer-reviewed journal article
**Caramanica et al., 2015**	Posttraumatic Stress Disorder after Hurricane Sandy among Persons Exposed to the 9/11 Disaster.	no data on internally displaced individuals
**Carmassi et al., 2020**	PTSD in the aftermath of a natural disaster: What we learned from the Pisa-L'Aquila Collaboration Project	no primary research
**Carrillo et al., 2022**	Physical and mental health outcomes of displacement: Comparisons of mental health, hair cortisol, and non-communicable disease risk indicators due to displacement in Vanuatu	no peer-reviewed journal article
**Castaño et al., 2019**	Analysis and profiles of drug users in a population of victims of forced displacement in Colombia	sample duplicate
**Castaño et al., 2018**	Mental disorders and drug use in victims of the armed conflict in three cities in Colombia	not focusing on point prevalences
**Chao, 2016**	Outdoor activities and depressive symptoms in displaced older adults following natural disaster: community cohesion as mediator and moderator.	no validated instrument based on DSM/ICD
**Chao, 2017**	Social support, coping strategies and their correlations with older adults' relocation adjustments after natural disaster.	no validated instrument based on DSM/ICD
**Charlson et al., 2012**	Predicting the impact of the 2011 conflict in Libya on population mental health: PTSD and depression prevalence and mental health service requirements	no primary research
**Chen et al., 2011**	Risk factors for PTSD after Typhoon Morakot among elderly people in Taiwanese aboriginal communities	N < 100
**Cheng et al., 2015**	Depression and posttraumatic stress disorder in temporary settlement residents 1 year after the Sichuan earthquake.	not focusing on point prevalences
**Cherry et al., 2015**	Long-term psychological outcomes in older adults after disaster: Relationships to religiosity and social support	no data on internally displaced individuals
**Chiba et al., 2024**	Associations between housing and psychological damage by earthquake and modifiable risk factors for dementia in general older adults: Tohoku Medical Megabank community-based cohort study.	no validated instrument based on DSM/ICD
**Chikovani et al., 2015**	Health Service Utilization for Mental, Behavioural and Emotional Problems among Conflict-Affected Population in Georgia: A Cross-Sectional Study	sample duplicate
**Chukwuorji et al., 2019**	Event centrality influences posttraumatic stress disorder symptoms via core beliefs in internally displaced older adults.	not focusing on point prevalences
**Chukwuorji et al., 2018**	Prolonged grief disorder symptoms in bereaved internally displaced Tiv persons in Nigeria: Associations with rumination, rebirth concerns and gender	not focusing on point prevalences

**Comellas et al., 2015**	Patterns of somatic distress among conflict-affected persons in the Republic of Georgia.	sample duplicate
**Corley et al., 2022**	Mental health effects of hurricane sandy on older adults	sample duplicate
**Coutinho et al., 1996**	Minor psychiatric morbidity and internal migration in Brazil.	no data on internally displaced individuals
**Dahl et al., 1998**	Traumatic events and predictive factors for posttraumatic symptoms in displaced Bosnian women in a war zone	no validated instrument based on DSM/ICD
**David et al., 2021**	Gender and Place of Settlement as Predictors of Perceived Social Support, PTSD, and Insomnia among Internally Displaced Adolescents in North-East Nigeria	not focusing on adults
**de la Fuente, 1990**	The mental health consequences of the 1985 earthquakes in Mexico	no validated instrument based on DSM/ICD
**DeSalvo et al., 2007**	Symptoms of posttraumatic stress disorder in a New Orleans workforce following Hurricane Katrina.	no data on internally displaced individuals
**Dodeler & Tarquinio, 2008**	Évaluation à long terme de l'impact psychologique et social des affaissements miniers d'Auboué sur les sinistrés ou populations déplacées = Long term evaluation of psychological and social consequences on victims and relocated people after mining subsidence in Auboué	no data on internally displaced individuals
**Doherty et al., 2019**	Prevalence of mental disorders and epidemiological associations in post-conflict primary care attendees: a cross-sectional study in the Northern Province of Sri Lanka.	sample not representative
**Dolezal et al., 2021**	Differences in posttraumatic and psychosocial outcomes among refugees, asylum seekers, and internally displaced persons	N < 100
**Ehsan et al., 2019**	Trauma of terror and displacement: A narrative analysis of mental health of women IDPS in KPK (Pakistan)	no validated instrument based on DSM/ICD
**Elhabiby et al., 2015**	Psychiatric disorders among a sample of internally displaced persons in South Darfur	N < 100
**Elklit, 2007**	Psychological consequences of a firework factory disaster in a local community	no data on internally displaced individuals
**Ertl et al., 2014**	The challenge of living on: Psychopathology and its mediating influence on the readjustment of former child soldiers	no data on internally displaced individuals
**Eustace et al., 1999**	Cyclone Bola: A study of the psychological after-effects	no data on internally displaced individuals
**Eze et al., 2022**	Locating event centrality in associations of emotion regulation with posttraumatic stress disorder symptoms and posttraumatic growth in emerging adults.	not focusing on point prevalences
**Fitzgerald et al., 2020**	Cross sectional analysis of depression amongst Australian rural business owners following cyclone-related flooding.	no validated instrument based on DSM/ICD
**Fitzpatrick, 2021**	Post-traumatic stress symptomatology and displacement among Hurricane Harvey survivors.	no validated instrument based on DSM/ICD
**Funakubo et al., 2021**	Association between Psychosocial Factors and Oral Symptoms among Residents in Fukushima after the Great East Japan Earthquake: A Cross-Sectional Study from the Fukushima Health Management Survey.	no data on internally displaced individuals
**Gaboury et al., 2023**	Posttraumatic growth and its relationship with posttraumatic stress symptoms following wildfires: A longitudinal study	not focusing on point prevalences

**Gao et al., 2021**	An epidemiological survey of mental disorders 5 years after the Lushan earthquake in Ya'an, China.	no validated instrument based on DSM/ICD
**García et al., 2024**	The impact of cultural stress on family functioning among Puerto Rican displaced families and the effect on mental health.	not focusing on point prevalences
**Gebreyesus et al., 2023**	Immediate health and economic impact of the Tigray war on internally displaced persons and hosting households.	sample duplicate
**Généreux et al., 2019**	Monitoring Adverse Psychosocial Outcomes One and Two Years After the Lac-Mégantic Train Derailment Tragedy (Eastern Townships, Quebec, Canada).	no data on internally displaced individuals
**Ghafouri et al., 2024**	Mental health in genocide: Balancing between posttraumatic distress and growth among displaced Yazidi mothers.	not focusing on point prevalences
**Gichunge et al., 2020**	Predictors of social support, physical health and mental health among food insecure internally displaced persons in Turkana, Kenya.	no validated instrument based on DSM/ICD
**Goessmann et al., 2020**	Association of War-Related and Gender-Based Violence With Mental Health States of Yazidi Women.	sample duplicate
**Gonzalez et al., 2024**	Risk and protective factors associated with substance use among Puerto Rican youths after Hurricane María: a cross-sectional study.	not focusing on adults
**Goto et al., 2002**	PTSD, depression and help-seeking patterns following the Miyake Island volcanic eruption	not focusing on point prevalences
**Gülşen et al., 2010**	The impact of forced migration on mental health: A comparative study on posttraumatic stress among internally displaced and externally migrated Kurdish women	no data on internally displaced individuals (?)
**Gusak et al., 2024**	Exploring prevalence and factors associated with postpartum depression among Ukrainian women.	N < 100
**Hagiwara et al., 2017**	Association Between Sleep Disturbance and New-onset Subjective Shoulder Pain in Survivors of the Great East Japan Earthquake: A Prospective Cohort Study in Miyagi Prefecture	sample duplicate
**Harigane et al., 2023**	Posttraumatic stress response following the loss of significant close others in the Great East Japan Earthquake: Fukushima Health Management Survey.	sample duplicate
**Harville et al., 2015**	When is exposure to a natural disaster traumatic? Comparison of a trauma questionnaire and disaster exposure inventory.	no data on internally displaced individuals
**Havenaar et al., 1996**	Mental health problems in the Gomel region (Belarus): an analysis of risk factors in an area affected by the Chernobyl disaster.	sample not representative
**Hayashi et al., 2021**	Association between post-traumatic stress disorder symptoms and bone fractures after the Great East Japan Earthquake in older adults: a prospective cohort study from the Fukushima Health Management Survey.	no data on internally displaced individuals
**Heeke et al., 2017**	Conflict-related trauma and bereavement: exploring differential symptom profiles of prolonged grief and posttraumatic stress disorder.	sample duplicate
**Hikichi et al., 2021**	Six-year follow-up study of residential displacement and health outcomes following the 2011 Japan Earthquake and Tsunami.	no validated instrument based on DSM/ICD
**Hirai et al., 2022**	Psychological burden predicts new-onset diabetes in men: A longitudinal observational study in the Fukushima Health Management Survey after the Great East Japan earthquake.	sample duplicate
**Hirai et al., 2021**	Suboptimal diabetic control and psychological burden after the triple disaster in Japan: the Fukushima Health Management Survey.	no data on internally displaced individuals

**Hossain et al., 2021**	Effects of riverbank erosion on mental health of the affected people in Bangladesh.	no useful comparison data
**Hunt & Gakenyi, 2005**	Comparing refugees and nonrefugees: the Bosnian experience.	no validated instrument based on DMS/ICD
**Hyland et al., 2023**	The structure of psychological distress in the aftermath of the 2023 Turkey/Syria earthquakes	not focusing on point prevalences
**Hyre et al., 2007**	Prevalence and predictors of posttraumatic stress disorder among hemodialysis patients following Hurricane Katrina.	sample not representative
**Ibrahim et al., 2018**	The validity of Posttraumatic Stress Disorder Checklist for DSM-5 (PCL-5) as screening instrument with Kurdish and Arab displaced populations living in the Kurdistan region of Iraq	not focusing on point prevalences
**Idemudia et al., 2020**	Pre-displacement stressors, psychological morbidity, and PTSD symptoms among displaced women by Boko-Haram in North-East Nigeria: The buffering roles of psychological capital	no validated instrument based on DSM/ICD (?)
**Idemudia et al., 2023**	Reciprocal Association between Psychological Distress and PTSD and Their Relationship with Pre-Displacement Stressors among Displaced Women	sample duplicate
**Ishii et al., 2022**	Postpartum Mental Health of Mothers in Fukushima: Insights From the Fukushima Health Management Survey's 8-year Trends.	no validated instrument based on DSM/ICD
**Ishiki et al., 2016**	Changes in Cognitive Functions in the Elderly Living in Temporary Housing after the Great East Japan Earthquake.	not focusing on point prevalences
**Ishikuro et al., 2022**	Families' Health after the Great East Japan Earthquake: Findings from the Tohoku Medical Megabank Project Birth and Three-Generation Cohort Study	no data on internally displaced individuals (?)
**Iwasa et al., 2016**	Psychometric Evaluation of the Japanese Version of the Posttraumatic Stress Disorder Checklist in Community Dwellers Following the Fukushima Daiichi Nuclear Power Plant Incident: The Fukushima Health Management Survey	no data on internally displaced individuals
**Jaddoue, 2024**	Assessment of the Health Status of Internally Displaced Persons (IDPs) in Camps and Sectors in Ameriyat Al-Samoud District in Al-Anbar Governorate	sample not representative
**Jayawickreme et al., 2017**	Measuring Depression in a Non-Western War-Affected Displaced Population: Measurement Equivalence of the Beck Depression Inventory.	sample not representative
**Jeftić et al., 2023**	Connection between the COVID-19 pandemic, war trauma reminders, perceived stress, loneliness, and PTSD in Bosnia and Herzegovina	N < 100
**Jiang et al., 2016**	Relationships between sleep problems and psychiatric comorbidities among China's Wenchuan earthquake survivors remaining in temporary housing camps	sample not representative
**Kaiser et al., 2020**	Mental health and psychosocial support needs among people displaced by Boko Haram in Nigeria.	N < 100
**Kaneda et al., 2024**	2024 Noto Peninsula Earthquake-elderly evacuees and mental health challenges.	no primary research
**Kang et al., 2023**	Disability and post-traumatic stress symptoms in the Ukrainian General Population during the 2022 Russian invasion	sample duplicate
**Kaplan et al., 2024**	The impact of earthquakes on women: assessing women's mental health in aftermath of the Kahramanmaraş-centred earthquake in Türkiye.	no validated instrument based on DSM/ICD
**Kar et al., 2022**	Anxiety, Depression, and Post-traumatic Stress a month after 2019 Cyclone Fani in Odisha, India.	N < 100

**Karamustafalıoğlu et al., 2023**	Ten-Year Follow-Up of Earthquake Survivors: Long-Term Study on the Course of PTSD Following a Natural Disaster.	no data on internally displaced individuals
**Kawakami et al., 2020**	Onset and remission of common mental disorders among adults living in temporary housing for three years after the triple disaster in Northeast Japan: comparisons with the general population.	not focusing on point prevalences
**Khailenko et al., 2024**	Resilience, avoidant coping and post-traumatic stress symptoms among female Ukrainian refugees and internally displaced people.	not focusing on point prevalences
**Kira et al., 2022**	The etiology of complex PTSD in the COVID-19 and continuous traumatic stressors era: A test of competing and allied models	sample duplicate
**Kizilhan et al., 2024**	Suicidality after the genocide against the Yazidi in Iraq in 2014	sample not representative
**Koch et al., 2015**	War, Displacement and Post War Period as potential Influencing Factors for mental Health in the Old Age	no peer-reviewed journal article
**Kohrt et al., 2012**	Political violence and mental health in Nepal: prospective study	N < 100
**Kongshøj et al., 2023**	Is young age a risk factor for PTSD? Age differences in PTSD-symptoms after Hurricane Florence	not focusing on point prevalences
**Korostiy et al., 2016**	Mental health of internally displaced persons in Ukraine	no peer-reviewed journal article
**Kouadio et al., 2012**	Military and political crises and the psycho-physical health of internally displaced persons (IDPs): the case of Côte d'Ivoire	no validated instrument based on DSM/ICD
**Kozhyna et al., 2018**	Depressive disorders in internally displaced persons	no peer-reviewed journal article
**Kramer et al., 2002**	Community needs assessment of lower Manhattan residents following the World Trade Center attacks--Manhattan, New York City, 2001.	no data on internally displaced individuals
**Kukihara et al., 2014**	Trauma, depression, and resilience of earthquake/tsunami/nuclear disaster survivors of Hirono, Fukushima, Japan	no validated instrument based on DSM/ICD
**Kun et al., 2009**	Prevalence and risk factors for posttraumatic stress disorder: A cross-sectional study among survivors of the Wenchuan 2008 earthquake in China	sample duplicate
**Kuwert et al., 2007**	More than 60 years later: the mediating role of trauma and posttraumatic stress disorder for the association of forced displacement in world war II with somatization in old age.	sample duplicate
**La Greca et al., 2022**	Evacuation- and hurricane-related experiences, emotional distress, and their associations with mothers' health risk behaviors	not focusing on point prevalences
**La Greca et al., 2022**	The stress before the storm: Psychological correlates of hurricane-related evacuation stressors on mothers and children	not focusing on point prevalences
**Labarda et al., 2020**	Long-term displacement associated with health and stress among survivors of Typhoon Haiyan.	no useful comparison data
**Lagos-Gallego et al., 2017**	Posttraumatic stress disorder in internally displaced people of Colombia: An ecological study	no validated instrument based on DSM/ICD
**LaJoie et al., 2010**	Long-term effects of Hurricane Katrina on the psychological well-being of evacuees.	no validated instrument based on DSM/ICD
**Lebowitz et al., 2019**	Post-flood social support networks and morbidity in Jôsô City, Japan.	no validated instrument based on DSM/ICD

**Lee et al., 2010**	A Community Mental Health Survey and Relief Program in Taiwan after the Great Earthquake-Implementation, Clinical Observation and Evaluation	sample not representative
**Lee et al., 2003**	A field study of posttraumatic stress disorder in a community after Typhoon Rusa	no validated instrument based on DSM/ICD
**León-Giraldo et al., 2023**	Trastornos de salud mental en población desplazada por el conflicto en Colombia: Análisis comparado frente a la Encuesta Nacional de Salud Mental 2015 = Mental health disorders in population displaced by conflict in Colombia: Comparative analysis against the National Mental Health Survey 2015	no validated instrument based on DSM/ICD
**Letica-Crepulja et al., 2011**	Factors associated with posttraumatic stress disorder and depression in war-survivors displaced in Croatia.	no data on internally displaced individuals
**Li et al., 2023**	Rumination about the Russo-Ukrainian War and its related factors among individuals in Poland and Ukraine.	no data on internally displaced individuals
**Li et al., 2010**	PTSD in a huge temporary house community after Wenchuan earthquake, Sichuan, China	no peer-reviewed journal article
**Li et al., 2010**	Post-traumatic stress disorder and depression symptoms among earthquake victims from different communities 3 months after the earthquake	no validated instrument based on DSM/ICD
**Lima et al., 1987**	Screening for the psychological consequences of a major disaster in a developing country: Armero, Colombia	no validated instrument based on DSM/ICD
**Lima et al., 1988**	La atención primaria de salud mental en las víctimas del desastre de Armero, Colombia = Mental health primary care among victims of the mudslide disaster in Armero, Colombia	no validated instrument based on DSM/ICD
**Logie et al., 2020**	Contextual factors associated with depression among urban refugee and displaced youth in Kampala, Uganda: findings from a cross-sectional study.	no data on internally displaced individuals
**Lommen et al., 2009**	Psychosocial predictors of chronic post-traumatic stress disorder in Sri Lankan tsunami survivors	N < 100
**Lopes Cardozo et al., 2004**	Mental Health, Social Functioning, and Disability in Postwar Afghanistan	no data on internally displaced individuals
**Lotzin et al., 2023**	War-related stressors and ICD-11 (complex) post-traumatic stress disorders in Ukrainian students living in Kyiv during the Russian-Ukrainian war	insufficient information
**Löw et al., 2023**	Resource loss, coping strategies and post-traumatic stress disorder symptoms in survivors of the 2020 Croatia earthquake.	no data on internally displaced individuals
**Lowe et al., 2013**	Trajectories of psychological distress among low-income, female survivors of Hurricane Katrina.	no validated instrument based on DSM/ICD
**Lowe et al., 2013**	Immediate and Longer-Term Stressors and the Mental Health of Hurricane Ike Survivors	sample duplicate
**Luitel et al., 2013**	Conflict and mental health: a cross-sectional epidemiological study in Nepal	no data on internally displaced individuals
**Luo et al., 2022**	Cango Lyec (Healing the Elephant): Probable post-traumatic stress disorder (PTSD) and depression in Northern Uganda five years after a violent conflict.	no data on internally displaced individuals
**Madianos et al., 2011**	Posttraumatic stress disorders comorbid with major depression in West Bank, Palestine: a general population cross sectional study	not focusing on point prevalences
**Madianos et al., 2012**	Major depression across West Bank: a cross-sectional general population study.	sample duplicate

**Mahmood et al., 2022**	The mental health of forcibly displaced couples.	no data on internally displaced individuals
**Makhashvili et al., 2017**	Mental health conditions and co-morbidities among internally displaced populations (IDPs) in Ukraine	no peer-reviewed journal article
**Malamba et al., 2016**	"The Cango Lyec Project - Healing the Elephant": HIV related vulnerabilities of post-conflict affected populations aged 13-49 years living in three Mid-Northern Uganda districts.	sample duplicate
**Maltais et al., 2005**	Social support, coping and psychological health after a flood	no data on internally displaced individuals
**Mandic & Mihaljevic, 1993**	Psychologic state of displaced persons from East Slavonia	sample not representative
**Mao et al., 2022**	Post-Traumatic Stress Disorder, Major Depressive Disorder, and Wildfires: A Fifth-Year Postdisaster Evaluation among Residents of Fort McMurray.	no data on internally displaced individuals
**Mao et al., 2022**	One Year after the Flood: Prevalence and Correlates of Post-Traumatic Stress Disorder among Residents in Fort McMurray.	no data on internally displaced individuals
**Marshall et al., 2007**	Psychiatric disorders among adults seeking emergency disaster assistance after a wildland-urban interface fire.	sample not reptresentative
**Marshall et al., 2010**	All PTSD symptoms are highly associated with general distress: ramifications for the dysphoria symptom cluster.	not focusing on point prevalences
**Maruta et al., 2019**	Risk factors for mental health impairments in internally displaced persons	N < 100
**Math et al., 2008**	Comparative study of psychiatric morbidity among the displaced and non-displaced populations in the Andaman and Nicobar Islands following the tsunami.	no validated instrument based on DSM/ICD
**McGuire et al., 2018**	Social Support Moderates Effects of Natural Disaster Exposure on Depression and Posttraumatic Stress Disorder Symptoms: Effects for Displaced and Nondisplaced Residents.	sample duplicate
**Melese et al., 2024**	Symptoms of posttraumatic stress, anxiety, and depression, along with their associated factors, among Eritrean refugees in Dabat town, northwest Ethiopia, 2023.	no validated instrument based on DSM/ICD
**Mels et al., 2010**	The psychological impact of forced displacement and related risk factors on Eastern Congolese adolescents affected by war.	not focusing on adults
**Minakshi et al., 2020**	Mental Health Problems in Wake of Disaster: A Gendered Perspective	not focusing on point prevalences
**Miyaji et al., 2022**	Social Capital and Post-traumatic Stress Disorder among Heavy Rainfall and Flood Victims in Japan.	no validated instrument based on DSM/ICD
**Mollica et al., 1993**	The effect of trauma and confinement on functional health and mental health status of Cambodians living in Thailand-Cambodia border camps.	no data on internally displaced individuals
**Mordeno et al., 2017**	DSM-5-based latent PTSD models: Assessing structural relations with GAD in Filipino post-relocatees.	sample duplicate
**Mordeno et al., 2022**	The paradoxical effect of interpersonal support from the social media on the post-relocation adjustment difficulties among filipino typhoon survivors	sample duplicate
**Mordeno et al., 2016**	PTSD factor structure and relationship with self-construal among internally displaced persons	sample not representative
**Mortensen et al., 2009**	Physical and mental health status of Hurricane Katrina evacuees in Houston in 2005 and 2006.	no validated instrument based on DSM/ICD

**Mugisha et al., 2015**	Prevalence and factors associated with Posttraumatic Stress Disorder seven years after the conflict in three districts in northern Uganda (The Wayo-Nero Study)	no data on internally displaced individuals
**Mugisha et al., 2015**	Major depressive disorder seven years after the conflict in northern Uganda: burden, risk factors and impact on outcomes (The Wayo-Nero Study)	no data on internally displaced individuals
**Munro et al., 2017**	Effect of evacuation and displacement on the association between flooding and mental health outcomes: a cross-sectional analysis of UK survey data.	no validated instrument based on DSM/ICD
**Muraspahić et al., 2017**	Effect of Sanitary-Environmental Conditions of Diabetic Hypertension Incidence in Displaced Persons.	no validated instrument based on DSM/ICD
**Nakamura et al., 2020**	Sleep Disturbance of Evacuees in Minamisanriku Town after Great East Japan Earthquake: Risk Factors and Treatment.	no validated instrument based on DSM/ICD
**Najarian et al., 2017**	Effect of relocation after a natural disaster in Armenia: 20-year follow-up.	N < 100
**Newman et al., 2022**	The relationships between neighbourhood vacancy, probable PTSD, and health-related quality of life in flood-disaster-impacted communities	not focusing on point prevalences
**Nickerson et al., 2024**	Moral injury appraisals and complex PTSD in refugees: A longitudinal study	no data on internally displaced individuals
**Ng et al., 2017**	Posttraumatic stress disorder, trauma, and reconciliation in South Sudan.	no primary research
**Ng et al., 2022**	Development of the South Sudan Mental Health Assessment Scale.	not focusing on point prevalences
**Norberg et al., 2022**	How do non-catastrophic natural disasters impact middle-aged-to-older persons? Using baseline Canadian longitudinal study on aging data to explore psychological outcomes associated with the 2013 Calgary flood	no validated instrument based on DSM/ICD
**Norris et al., 2004**	Postdisaster PTSD over four waves of a panel study of Mexico's 1999 flood	no data on internally displaced individuals
**Nuttman-Shwartz et al., 2011**	Post-traumatic stress and growth following forced relocation	no data on internally displaced individuals
**Nzayisenga et al., 2022**	Patterns of distress and psychosocial support 2 years post-displacement following a natural disaster in a lower middle income country.	no validated instrument based on DSM/ICD
**Oe et al., 2016**	Three-year trend survey of psychological distress, post-traumatic stress, and problem drinking among residents in the evacuation zone after the Fukushima Daiichi Nuclear Power Plant accident [The Fukushima Health Management Survey].	no data on internally displaced individuals
**Oe et al., 2017**	Changes of posttraumatic stress responses in evacuated residents and their related factors: A 3-year follow-up study from the Fukushima Health Management Survey	no data on internally displaced individuals
**Oishi et al., 2021**	Risk Perception of Health Risks Associated with Radiation Exposure among Residents of Okuma, Fukushima Prefecture.	no data on internally displaced individuals
**Ojeahere et al., 2021**	Assessment of full and subsyndromal PTSD and quality of life of internally displaced older adults in northern Nigeria	not focusing on point prevalences
**Oren & Possick, 2010**	Is ideology a risk factor for PTSD symptom severity among Israeli political evacuees?	sample duplicate
**Oren & Possick, 2009**	Religiosity and posttraumatic stress following forced relocation	no data on internally displaced individuals

**Owusu et al., 2022**	Prevalence and determinants of generalized anxiety disorder symptoms in residents of Fort McMurray 12 months following the 2020 flooding	no data on internally displaced individuals
**Palace et al., 2024**	Mapping the factors behind ongoing war stress in Ukraine-based young civilian adults.	no data on internally displaced individuals
**Paranjothy et al., 2011**	Psychosocial impact of the summer 2007 floods in England.	insufficient information
**Parslow et al., 2006**	Associations of pre-trauma attributes and trauma exposure with screening positive for PTSD: analysis of a community-based study of 2,085 young adults.	no data on internally displaced individuals
**Pavlenko et al., 2024**	War impact on Ukrainian university women: Does location status effect depression and quality of life factors?	not focusing on point prevalences
**Pavlenko et al., 2023**	Ukrainian 'help' profession women: War and location status impact on well-being	no validated instrument based on DSM/ICD
**Peng et al., 2023**	Assessment of post-traumatic stress disorder (PTSD) in elderly survivors of the Wenchuan earthquake: A cross-sectional study.	sample not representative
**Pham et al., 2021**	Association between distress and displacement settings: a cross-sectional survey among displaced Yazidis in northern Iraq.	no validated instrument based on DSM/ICD
**Pham et al., 2004**	Trauma and PTSD symptoms in Rwanda: implications for attitudes toward justice and reconciliation.	no data on internally displaced individuals
**Pineros-Leano et al., 2023**	Technology-based communication among Hurricane Maria survivors in the United States: a trans-territorial lens.	no validated instrument based on DSM/ICD
**Policastro et al., 2023**	Adaptation, Validity, and Reliability of the Patient Health Questionnaire (PHQ-9) in the Kurdistan Region of Iraq.	no useful comparison data
**Popovski & Naumova, 2008**	Trauma and posttraumatic stress in war IDPs in Macedonia	N < 100
**Rahimi et al., 2023**	Post-traumatic stress disorder (PTSD) probability among parents who live in Kandahar, Afghanistan and lost at least a child to armed conflict.	no data on internally displaced individuals
**Rahman & Gain, 2020**	Adaptation to river bank erosion induced displacement in Koyra Upazila of Bangladesh	no validated instrument based on DSM/ICD
**Raker et al., 2019**	Twelve years later: The long-term mental health consequences of Hurricane Katrina.	no validated instrument based on DSM/ICD
**Rasheed et al., 2022**	Withstanding psychological distress among internally displaced Yazidis in Iraq: 6 years after attack by the Islamic State of Iraq and the Levant.	no validated instrument based on DSM/ICD
**Reppesgaard, 1997**	Studies on psychosocial problems among displaced people in Sri Lanka	no validated instrument based on DSM/ICD
**Roberts et al., 2009**	Factors associated with the health status of internally displaced persons in northern Uganda.	sample duplicate
**Roberts et al., 2019**	Mental health care utilisation among internally displaced persons in Ukraine: results from a nation-wide survey.	sample duplicate
**Roberts et al., 2013**	Tobacco use and nicotine dependence among conflict-affected men in the Republic of Georgia.	sample duplicate
**Roberts et al., 2009**	Post-conflict mental health needs: A cross-sectional survey of trauma, depression and associated factors in Juba, Southern Sudan	no data on internally displaced individuals

**Roberts et al., 2014**	Individual and community level risk-factors for alcohol use disorder among conflict-affected persons in Georgia	sample duplicate
**Roberts et al., 2017**	Access to mental health services among internally displaced persons in Ukraine: Results from a nationwide survey	sample duplicate
**Rockers et al., 2010**	Village characteristics associated with posttraumatic stress symptoms in postconflict Liberia.	not focusing on point prevalences
**Rodriguez et al., 2022**	Community Assessment for Public Health Emergency Response (CASPER) Following Hurricane Michael, Bay and Gulf Counties, Florida, 2019.	not focusing on point prevalences
**Rodríguez-Pérez et al., 2024**	Health impact of the Tajogaite volcano eruption in La Palma population (ISVOLCAN study): rationale, design, and preliminary results from the first 1002 participants.	no validated instrument based on DSM/ICD
**Rofo et al., 2023**	Prevalence and risk factors of posttraumatic stress symptoms among Internally Displaced Christian couples in Erbil, Iraq.	sample not representative
**Ruggiero et al., 2012**	Mental health outcomes among adults in Galveston and Chambers counties after Hurricane Ike.	N < 100
**Salah et al., 2015**	Social phobia among long-term internally displaced persons: An epidemiological study of adults in two internally displaced person settlements in Sudan.	sample duplicate
**Şalcioğlu et al., 2008**	Psychosocial determinants of relocation in survivors of the 1999 earthquake in Turkey	sample not representative
**Sanguanklin et al., 2014**	Effects of the 2011 flood in Thailand on birth outcomes and perceived social support.	no validated instrument based on DSM/ICD
**Sanhori et al., 2020**	Changes in prevalence of mental disorders among internally displaced persons in central Sudan: A 1-year follow-up study	sample duplicate
**Sato et al., 2016**	Factors relating to the mental health of women who were pregnant at the time of the Great East Japan earthquake: Analysis from month 10 to month 48 after the earthquake	no validated instrument based on DSM/ICD
**Sattler et al., 2014**	Indian Ocean tsunami: Relationships among posttraumatic stress, posttraumatic growth, resource loss, and coping at 3 and 15 months	no validated instrument based on DSM/ICD
**Saxon et al., 2017**	Coping strategies and mental health outcomes of conflict-affected persons in the Republic of Georgia.	sample duplicate
**Scholte et al., 2004**	Mental health symptoms following war and repression in eastern Afghanistan.	no data on internally displaced individuals
**Schwartz et al., 2019**	Examining Associations Between Hurricane Sandy Exposure and Posttraumatic Stress Disorder by Community of Residence.	sample duplicate
**Schwartz et al., 2018**	Displacement during Hurricane Sandy: The impact on mental health.	no data on internally displaced individuals
**Schwartz et al., 2017**	Displacement and mental health after natural disasters	no primary research
**Schwartz et al., 2024**	After Hurricane Maria: Effects of disaster trauma on Puerto Rican survivors on the US mainland	not focusing on point prevalences
**Scull, 2015**	Forgiveness, revenge, and adherence to Islam as moderators for psychological wellbeing and depression among survivors of the 1990 Iraqi invasion of Kuwait	no data on internally displaced individuals
**Seidi et al., 2023**	Mental health status of internally displaced persons in the Garmian region of Kurdistan, Iraq: a cross-sectional survey.	N < 100

**Seldomridge et al., 2024**	Adherence to Gender Roles on PTSD Symptoms of Hurricane Harvey Survivors	not focusing on point prevalences
**Self-Brown et al., 2014**	Maternal posttraumatic stress disorder symptom trajectories following Hurricane Katrina: An initial examination of the impact of maternal trajectories on the well-being of disaster-exposed youth.	not focusing on point prevalences
**Sezgin & Punamäki, 2016**	Women’s disaster-related mental health: The decision to leave or to stay after an earthquake	not focusing on point prevalences
**Sharma et al., 2022**	The measurement of war-related trauma amongst internally displaced men and women in South Sudan: Psychometric analysis of the Harvard Trauma Questionnaire.	not focusing on point prevalences
**Shevlin et al., 2018**	A comparison of DSM-5 and ICD-11 PTSD prevalence, comorbidity and disability: an analysis of the Ukrainian Internally Displaced Person's Mental Health Survey.	sample duplicate
**Shultz et al., 2019**	A pilot study of a stepped-care brief intervention to help psychologically-distressed women displaced by conflict in Bogotá, Colombia	sample not representative
**Shvartsur & Savitsky, 2024**	Civilians under missile attack: post-traumatic stress disorder among the Jewish and Bedouin population of Southern Israel	no data on internally displaced individuals
**Siriwardhana et al., 2015**	Dynamics of resilience in forced migration: a 1-year follow-up study of longitudinal associations with mental health in a conflict-affected, ethnic Muslim population.	sample duplicate
**Siriwardhana et al., 2013**	Prolonged internal displacement and common mental disorders in Sri Lanka: The COMRAID study	sample duplicate
**Sodeyama et al., 2022**	The Mental Health of Long-Term Evacuees outside Fukushima Prefecture after the Great East Japan Earthquake	no validated instrument based on DSM/ICD
**Sodeyama et al., 2022**	A Comparison of Mental Health among Earthquake, Tsunami, and Nuclear Power Plant Accident Survivors in the Long Term after the Great East Japan Earthquake	no validated instrument based on DSM/ICD
**Somasundaram et al., 2023**	Effect of daily stressors and collective efficacy on post-traumatic stress symptoms among internally displaced persons in post-war northern Sri Lanka.	no validated instrument based on DSM/ICD
**Somer et al., 2009**	Israeli civilians under heavy bombardment: prediction of the severity of post-traumatic symptoms.	no data on internally displaced individuals
**Sryh & Ozcebe, 2020**	Mental health and quality of life assessment among adult internally displaced persons, Tripoli Libya	no peer-reviewed journal article
**Stukova et al., 2023**	Mental health and associated risk factors of Puerto Rico Post-Hurricane María.	not focusing on point prevalences
**Tadesse et al., 2024**	Magnitude and factors associated with post-traumatic stress disorder among war-affected internally displaced people in northwest Ethiopia, 2022.	sample duplicate
**Taha & Slewa-Younan, 2020**	Measures of depression, generalized anxiety, and posttraumatic stress disorders amongst Yazidi female survivors of ISIS slavery and violence	sample not representative
**Taha et al., 2021**	Posttraumatic stress disorder correlates among internally displaced Yazidi population following Islamic state of Iraq and Syria attacks in Iraq	sample duplicate
**Takahashi et al., 2020**	Effects of Psychological and Lifestyle Factors on Metabolic Syndrome Following the Fukushima Daiichi Nuclear Power Plant Accident: The Fukushima Health Management Survey	sample duplicate
**Takahashi et al., 2023**	Impact of Lifestyle and Psychosocial Factors on the Incidence of Hepatobiliary Enzyme Abnormalities After the Great East Japan	sample duplicate

	Earthquake: Seven-Year Follow-up of the Fukushima Health Management Survey.	
**Takaoka et al., 2018**	The association of posttraumatic stress disorder risk with help-seeking behavior: A cross-sectional study of earthquake and tsunami survivors in Japan	no validated instrument based on DSM/ICD
**Tally et al., 2013**	The impact of the San Diego wildfires on a general mental health population residing in evacuation areas.	no validated instrument based on DSM/ICD
**Tamayo Martínez et al., 2016**	Mental Problems, Mood and Anxiety Disorders in The Population Displaced by Violence in Colombia; Results of The National Mental Health Survey 2015.	not focusing on point prevalences
**Tanaka et al., 2019**	Effect of Pets on Human Behavior and Stress in Disaster.	no validated instrument based on DSM/ICD
**Tanaka & Takagi**	A study of the victims in the temporary housing built outside of the stricken disaster area of the great Hanshin-Awaji earthquake I: The impact on physical and mental health of the people in the temporary housing a year after the earthquake	no validated instrument based on DSM/ICD
**Tay et al., 2017**	The factor structures and correlates of PTSD in post-conflict Timor-Leste: An analysis of the Harvard Trauma Questionnaire	insufficient information
**Tezuka et al., 2021**	Synergistic Effect of History of Cardiovascular Disease and Mental Distress on Post-Traumatic Stress Disorder after the Great East Japan Earthquake: The Fukushima Health Management Survey.	sample not representative
**Thapa et al., 2012**	Perceived needs, self-reported health and disability among displaced persons during an armed conflict in Nepal.	sample duplicate
**Thienkrua et al., 2006**	Symptoms of Posttraumatic Stress Disorder and Depression Among Children in Tsunami-Affected Areas in Southern Thailand	not focusing on adults
**Thomas et al., 2022**	Displacement-related stressors in a Sri Lankan war-affected community: Identifying the impact of war exposure and ongoing stressors on trauma symptom severity	sample not representative
**Thompson et al., 2015**	Stress and cortisol in disaster evacuees: an exploratory study on associations with social protective factors.	N < 100
**Toyabe et al., 2006**	Impaired psychological recovery in the elderly after the Niigata-Chuetsu Earthquake in Japan:a population-based study.	no validated instrument based on DSM/ICD
**Tracy et al., 2011**	Differences in the determinants of posttraumatic stress disorder and depression after a mass traumatic event	no data on internally displaced individuals
**Tsujiuchi, 2021**	Post-traumatic stress due to structural violence after the Fukushima Disaster	no validated instrument based on DSM/ICD
**Tucker et al., 2008**	Katrina survivors relocated to Oklahoma: A tale of two cities	N < 100
**Turnip et al., 2010**	The mental health of populations directly and indirectly exposed to violent conflict in Indonesia.	no useful comparison data
**Turnip et al., 2016**	Predicting positive mental health in internally displaced persons in Indonesia: the roles of economic improvement and exposure to violent conflict.	no validated instrument based on DSM/ICD
**Tuval-Mashiach & Dekel, 2012**	Preparedness, ideology, and subsequent distress: Examining a case of forced relocation	no data on internally displaced individuals
**Ueda et al., 2022**	A Six-Year Prospective Study on Problem Drinking among Evacuees of the Great East Japan Earthquake: The Fukushima Health Management Survey.	no data on internally displaced individuals

**Ueda et al., 2019**	Risk Factors for Problem Drinking among Evacuees in Fukushima following the Great East Japan Earthquake: The Fukushima Health Management Survey.	no data on internally displaced individuals
**Uemura et al., 2016**	Association between psychological distress and dietary intake among evacuees after the Great East Japan Earthquake in a cross-sectional study: the Fukushima Health Management Survey.	not focusing on point prevalences
**Ugbe et al., 2023**	Correlates of somatic symptom disorder among internally displaced persons in Ogoja displacement settlements, Nigeria: a cross-sectional study.	no validated instrument based on DSM/ICD
**Ursano et al., 2007**	Prevalence of and Sex Disparities in Posttraumatic Stress Disorder in an Internally Displaced Sri Lankan Population 6 Months After the 2004 Tsunami	no validated instrument based on DSM/ICD
**Usta et al., 2008**	Women, war, and violence: Surviving the experience	no validated instrument based on DSM/ICD
**van Berlaer et al., 2017**	Diagnoses, infections and injuries in Northern Syrian children during the civil war: A cross-sectional study.	not focusing on adults
**Vázquez et al., 2015**	La evacuación por inundaciones y su impacto en la percepción de riesgo y el estrés postraumático = Evacuation after flooding and its impact on risk perception and post-traumatic stress	N < 100
**Venger et al., 2024**	Structure and features of psychopathological symptoms in forced migrants and internally displaced persons.	insufficient information
**Vinck et al., 2007**	Exposure to war crimes and implications for peace building in Northern Uganda	no useful comparison data
**Viswanath et al., 2013**	Gender differences in the psychological impact of tsunami	no validated instrument based on DSM/ICD
**Viswanath et al., 2012**	Psychological impact of the tsunami on elderly survivors.	no validated instrument based on DSM/ICD
**Walling et al., 2020**	Neuropsychological Outcomes of Exposure to Hurricane Katrina and Relocation.	N < 100
**Wang et al., 2007**	Mental health service use among hurricane Katrina survivors in the eight months after the disaster.	no useful comparison data
**Wang et al., 2010**	Mental health change tendency of aging victims in Beichuan area 4-10 months after Wenchuan earthquake	no validated instrument based on DSM/ICD
**Watts et al., 2023**	Incidence and factors impacting PTSD following the 2005 Eyre Peninsula bushfires in South Australia - A 7 year follow up study.	N < 100
**Weems et al., 2007**	The psychosocial impact of Hurricane Katrina: Contextual differences in psychological symptoms, social support, and discrimination	not focusing on point prevalences
**Wendt et al., 2012**	How traumatized are the children of World War II? The relationship of age during flight and forced displacement and current posttraumatic stress symptoms	no validated instrument based on DSM/ICD
**Wu et al., 2012**	PTSD in relocated adolescents and social support and coping style after Senchuan earthquake	no peer-reviewed journal article
**Wu et al., 2016**	Posttraumatic stress disorder and posttraumatic growth coexistence and the risk factors in Wenchuan earthquake survivors.	no useful comparison data
**Xiao et al., 2023**	Assessment of Radiation Risk Perception and Interest in Tritiated Water among Returnees to and Evacuees from Tomioka Town within 20 km of the Fukushima Daiichi Nuclear Power Plant.	no useful comparison data

**Xiong et al., 2008**	Exposure to Hurricane Katrina, post-traumatic stress disorder and birth outcomes	no data on internally displaced individuals
**Xu et al., 2018**	Cumulative incidence of suicidal ideation and associated factors among adults living in temporary housing during the three years after the Great East Japan Earthquake.	not focusing on point prevalences
**Yabe et al., 2014**	Psychological distress after the Great East Japan Earthquake and Fukushima Daiichi Nuclear Power Plant accident: results of a mental health and lifestyle survey through the Fukushima Health Management Survey in FY2011 and FY2012.	no data on internally displaced individuals
**Yadav et al., 2019**	Post-Flood Rapid Needs Assessment in Srinagar City, Jammu and Kashmir State, India, September, 2014.	no validated instrument based on DSM/ICD
**Yagi et al., 2020**	Changes in drinking behavior among evacuees after the Fukushima Daiichi Nuclear Power Plant accident:the Fukushima Health Management Survey.	no data on internally displaced individuals
**Yamout & Chaaya, 2011**	Individual and collective determinants of mental health during wartime. A survey of displaced populations amidst the July-August 2006 war in Lebanon.	no useful comparison data
**Yazawa et al., 2023**	Post-Disaster Mental Health and Dietary Patterns among Older Survivors of an Earthquake and Tsunami.	no validated instrument based on DSM/ICD
**Yoshida et al., 2016**	Psychological distress of residents in Kawauchi village, Fukushima Prefecture after the accident at Fukushima Daiichi Nuclear Power Station: the Fukushima Health Management Survey.	no data on internally displaced individuals
**Yousef et al., 2021**	War-related trauma and post-traumatic stress disorder prevalence among Syrian university students.	no data on internally displaced individuals
**Yzermans et al., 2005**	Health problems of victims before and after disaster: a longitudinal study in general practice.	no mental disorder according to ICD/DSM
**Zahlawi et al., 2019**	Psychosocial support during displacement due to a natural disaster: relationships with distress in a lower-middle income country.	no validated instrument based on DSM/ICD
**Zerach & Tam, 2016**	The relationships between family functioning and attachment orientations to post-traumatic stress symptoms among young adults who were evacuated from Gaza Strip settlements as adolescents	no data on internally displaced individuals
**Zhabchenko et al., 2019**	Features of hormonal function in pregnant women - displaced persons	N < 100
**Zhabchenko et al., 2020**	Hormonal and metabolic features of the placental complex in pregnant women-displaced persons	N < 100
**Zhang et al., 2021**	Health-related quality of life among survivors in minority area 2 years after Jiuzhaigou earthquake: A cross-sectional study.	no data on internally displaced individuals
**Zhang et al., 2024**	Association of optimism, causal thinking, and karma beliefs with PTSD and depression 8 years after the tsunami in Sri Lanka	no validated instrument based on DSM/ICD
**Zhen et al., 2018**	Fear, negative cognition, and depression mediate the relationship between traumatic exposure and sleep problems among flood victims in China.	no validated instrument based on DSM/ICD
**Zhong et al., 2020**	Assessing the effectiveness and pathways of planned shelters in protecting mental health of flood victims in China	no data on internally displaced individuals
**OTHER SOURCES**		
**Alderman et al., 2013**	Assessment of the health impacts of the 2011 summer floods in Brisbane.	no data on internally displaced individuals

**Al Shawi, 2018**	Prevalence of posttraumatic stress disorders among sample of internally displaced persons in Iraq, a preliminary study	N < 100
**Asnakew et al., 2019**	Prevalence of post-traumatic stress disorder and associated factors among Koshe landslide survivors, Addis Ababa, Ethiopia: a community-based, cross-sectional study.	no data on internally displaced individuals
**Aurizki, 2020**	Factors associated with post-traumatic stress disorder (PTSD) following natural disaster among Indonesian elderly	N < 100
**Cairo et al. 2010**	The Prevalence of Posttraumatic Stress Disorder Among Adult Earthquake Survivors in Peru	N < 100
**Cao et al. 2017**	Support for the association between RORA gene polymorphisms and the DSM-5 posttraumatic stress disorder symptoms in male earthquake survivors in China.	no data on internally displaced individuals
**Cao et al., 2020**	A comparison of ICD- 11 and DSM-5 criteria for PTSD among a representative sample of Chinese earthquake survivors	no data on internally displaced individuals
**Chang et al., 2002**	Psychiatric morbidity and pregnancy outcome in a disaster area of Taiwan 921 earthquake.	not focusing on point prevalences
**Cherian et al., 2020**	Prevalence and factors associated with post-traumatic stress disorder among flood-affected adults in a panchayat in Ernakulam district in Kerala	N < 100
**Dell et al., 2024**	A machine learning approach using migration-related cultural stress to classify depression and post-traumatic stress disorder among hurricane survivors	sample duplicate
**Dell'Osso et al., 2012**	Post-traumatic stress spectrum in young versus middle-aged L'Aquila 2009 earthquake	no data on internally displaced individuals
**Dévieux et al., 2013**	Post-traumatic stress disorder symptomatology and alcohol use among HIV-seropositive adults in Haiti.	N < 100
**Goldmann et al., 2021**	Rapid Behavioral Health Assessment Post-disaster: Developing and Validating a Brief, Structured Module	N < 100
**Huang et al., 2010**	Epidemiological investigation on major depressive disorder in the most heavily damaged areas from Wenchuan earthquake in 2008.	no validated instrument based on DSM/ICD
**İlhan et al., 2023**	Prevalence and associated risk factors of post-traumatic stress disorder among survivors of the 2023 Turkey earthquake.	sample not representative
**Irniza et al., 2016**	The Association Between KAP on Disasters with Depression, GAD and PTSD Among Flood Victims	N < 100
**Jha, 2017**	Identification and treatment of Nepal 2015 earthquake survivors with posttraumatic stress disorder by nonspecialist volunteers: An exploratory cross-sectional study	no data on internally displaced individuals
**Jin., 2014**	Posttraumatic Stress Disorder and Posttraumatic Growth Among Adult Survivors of Wenchuan Earthquake After 1 Year: Prevalence and Correlate	no data on internally displaced individuals
**Karamustafalioglu et al., 2006**	Natural course of posttraumatic stress disorder: a 20-month prospective study of Turkish earthquake survivors.	no data on internally displaced individuals
**Kiliç & Ulusoy, 2003**	Psychological effects of the November 1999 earthquake in Turkey: an epidemiological study.	no data on internally displaced individuals
**Lai et al., 2015**	Hurricane katrina: maternal depression trajectories and child outcomes.	no data on internally displaced individuals
**Laor et al., 1996**	Israeli pre- schoolers under Scud missile attacks: a developmental perspective on risk-modifying factors.	N < 100

**Lopes Cardozo et al., 2004**	Karenni refugees living in Thai-Burmese border camps: traumatic experiences, mental health outcomes, and social functioning.	no data on internally displaced individuals
**Lu et al., 2020**	Prevalence and influencing factors of post-traumatic stress disorder among survivors in the hard-hit areas ten years after the Wenchuan earthquake: A cross-sectional study	no data on internally displaced individuals
**Mason et al., 2010**	The psychological impact of exposure to floods.	no data on internally displaced individuals
**Musau et al., 2018**	The prevalence of post-traumatic stress disorder (PTSD) among internally displaced persons (IDPs) in Maai Mahiu Camp in Nakuru County, Kenya.	sample not representative
**Nillni et al., 2013**	Unique and related predictors of major depressive disorder, posttraumatic stress disorder, and their comorbidity after Hurricane Katrina.	sample duplicate
**Norris et al., 2001**	Sex differences in symptoms of posttraumatic stress: Does culture play a role?	no data on internally displaced individuals
**Önder, 2006**	Prevalence of psychiatric disorders three years after the 1999 earthquake in Turkey: Marmara Earthquake Survey (MES)	no data on internally displaced individuals
**Qu et al., 2012**	The impact of the catastrophic earthquake in China's Sichuan province on the mental health of pregnant women.	no data on internally displaced individuals
**Qu et al., 2012**	Posttraumatic stress disorder and depression among new mothers at 8 months later of the 2008 Sichuan earthquake in China.	no data on internally displaced individuals
**Ramirez et al., 2016**	Afectaciones psicológicas, estrategias de afrontamiento y niveles de resiliencia de adultos expuestos al conflicto armado en Colombia [Psychological Affectations, Coping Strategies and Resilience Levels of Adults Exposed to the Armed Conflict in Colombia].	no data on internally displaced individuals
**Ramírez-Giraldo et al., 2017**	Trastornos de ansiedad y del estado de ánimo en personas víctimas del conflicto armado en Colombia: El caso de Chengue y de Libertad [Anxiety and mood disorders in victims of the armed conflict in Colombia: The case of Chengue and Libertad].	N < 100
**Rashid et al., 2020**	Post-traumatic stress disorder and association with low birth weight in displaced population following conflict in malakand division, Pakistan: a case control study.	sample not representative
**Sato, 2020**	Postdisaster Changes in Social Capital and Mental Health: A Natural Experiment From the 2016 Kumamoto Earthquake	sample duplicate
**Savron et al. 2000**	Caratteristiche psicologiche in soggetti con disturbo post-traumatico da stress cittime del terremoto del 1997 nella Regione Marche	N < 100
**Shore, 1989**	Community patterns of posttraumatic stress disorder	not focusing on point prevalences
**Taru et al., 2018**	Posttraumatic stress disorder among internally displaced victims of Boko Haram terrorism in north-eastern Nigeria	sample not representative
**Zenker, 2024**	Always on my mind: indications of post-traumatic stress disorder among those affected by the 2021 flood event in the Ahr valley, Germany	N < 100
**Zenker, 2025**	Die Auswirkungen des Hochwassers 2021 auf die mentale Gesundheit der Betroffenen	N < 100

Supplement 6: Results of moderator and subgroup analyses

	PTSD				MDD				GAD			
	**k**	**Prevalence (95% CI)**	**I^2**	**OR (95% CI)**	**k**	**Prevalence (95% CI)**	**I^2**	**OR (95% CI)**	**k**	**Prevalence (95% CI)**	**I^2**	**OR (95% CI)**
**Gender**				1.42 (1.11-1.82)				1.45 (0.67-3.13)				-
male	30	37.4% (28.1-47.7)	97.1%		4	67.3% (32.0-90.0)	99.5%		3	-	-	
female	33	47.7% (39.0-56.5)	98.1%		6	66.1% (39.4-85.5)	99.5%		4	39.1% (30.0-49.0)	97.1%	
	**k**	**Prevalence (95% CI)**	**I^2**	$p_{difference}$	**k**	**Prevalence (95% CI)**	**I^2**	$p_{difference}$	**k**	**Prevalence (95% CI)**	**I^2**	$p_{difference}$
**Displacement cause**				.0029				.3415				-
armed conflict	52	46.3% (38.9-53.9)	98.7%		24	35.2% (24.4-47.8)	99.4%		18	32.4% (22.0-44.8)	99.5%	
natural disaster	42	31.8% (26.2-37.9)	98.9%		11	28.3% (20.8-37.2)	96.8%		3	-	-	
**Income status**				.1954				.0469				.5030
low	27	42.1% (35.5-48.9)	97.4		12	53.4% (36.7-69.4)	99.4%		6	36.6% (18.8-58.9)	99.4%	
lower middle	33	38.2% (29.5-47.6)	99.4		11	27.0% (14.1-45.6)	99.3%		10	27.4% (13.9-46.8)	99.5%	
upper middle	17	41.4% (29-8-54.0)	98.0		5	39.1% (24.5-56.0)	99.1%		3	-	-	
high	13	29.0% (20.6-39.2)	95.9		9	24.6% (14.4-38.7)	97.3%		3	-	-	

Supplement 7: Overview of mental disorders investigated in <4 studies

Mental Disorder	Study	N	Prevalence
**Agoraphobia**	Banal (2010)	600	3.7%
	Salah (2013)	1876	6.8%
	Tekeli-Yesil (2018)	244	38.9%
**Anorexia**	Banal (2010)	600	0%
**Antisocial personality disorder**	Salah (2013)	1876	0.9%
**Bulimia nervosa**	Banal (2010)	600	0%
**Bipolar disorder**	Howard (1999)	351	0.3%
**cPTSD***	Karatzias (2023)	501	16.6%
	Kira (2023)	891	33.1%
	McGinty (2023)	2198	7.8%
**Dysthymia**	Banal (2010)	600	2.3%
	Howard (1999)	351	7.4%
	Salah (2013)	1876	19.9%
**Hypomanic episode**	Banal (2010)	600	0.8%
	Salah (2013)	1876	2.7%
**Manic episode**	Banal (2010)	600	1.5%
**Obsessive compulsory disorder**	Banal (2010)	600	2.5%
	Salah (2013)	1876	5.1%
**Prolonged grief disorder**	Ghaffari-Nejad (2007)	400	76.0%
	Heeke (2015)	295	29.5%
	Jann (2024)	199	56.3%
**Psychotic disorder**	Banal (2010)	600	0.5%
	Salah (2013)	1876	1.0%
**Substance use disorder**	Aluh (2024)	520	13.1%
	Banal (2010)	600	0.8%
	Salah (2013)	1876	1.0%
**Social phobia**	Banal (2010)	600	1.8%
	Salah (2013)	1876	14.2%

Supplement 8: Results for publication bias and sensitivity analyses

Funnel plot for PTSD outcome:

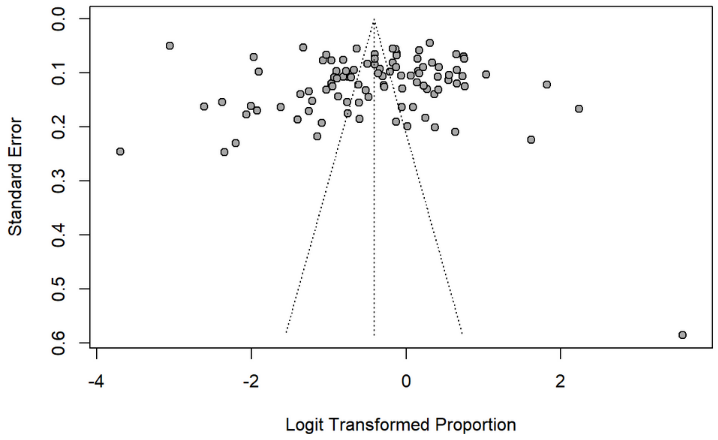

Funnel plot for depression outcome:

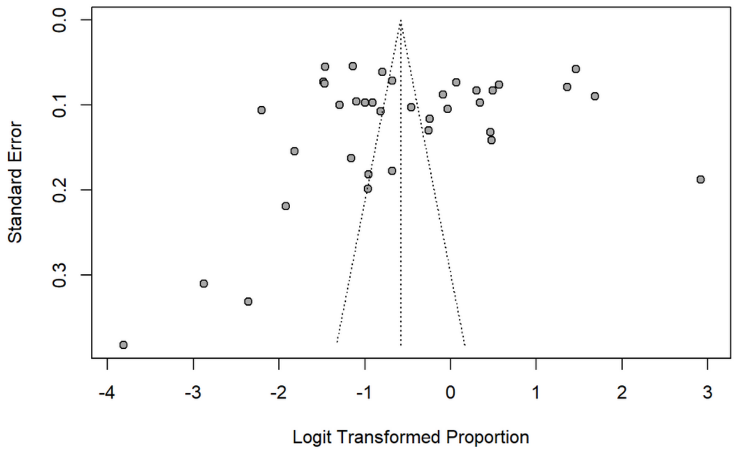

Funnel plot for generalised anxiety disorder outcome:

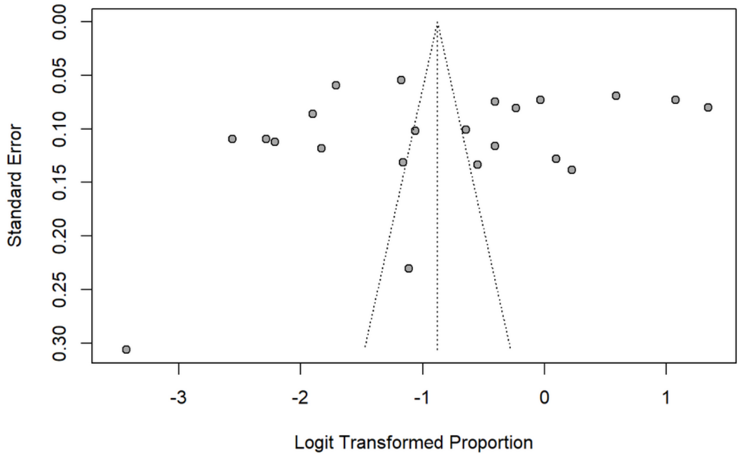

Outlier-adjusted analyses for PTSD:

```
Number of studies: k = 36
Number of observations: o = 17258
Number of events: e = 7066

                     proportion           95%-CI
Random effects model     0.4031 [0.3827; 0.4238]

Quantifying heterogeneity (with 95%-CIs):
 tau^2 = 0.0554; tau = 0.2355
 I^2 = 85.3% [80.6%; 88.9%]; H = 2.61 [2.27; 3.00]
```

Outlier-adjusted analyses for depression:

```
Number of studies: k = 15
Number of observations: o = 6661
Number of events: e = 2254

                     proportion           95%-CI
Random effects model     0.3386 [0.2987; 0.3809]

Quantifying heterogeneity (with 95%-CIs):
 tau^2 = 0.1169; tau = 0.3419
 I^2 = 91.6% [87.8%; 94.2%]; H = 3.45 [2.87; 4.16]
```

Outlier-adjusted analyses for generalised anxiety disorder:

```
Number of studies: k = 9
Number of observations: o = 5160
Number of events: e = 1611

                     proportion           95%-CI
Random effects model     0.3228 [0.2737; 0.3762]

Quantifying heterogeneity (with 95%-CIs):
 tau^2 = 0.1154; tau = 0.3398
 I^2 = 94.9% [92.2%; 96.6%]; H = 4.42 [3.58; 5.46]
```

Supplement 9: Reported adjusted odds ratio for comparisons IPD vs. non-displaced

Report	OR (95% CI)	Comparison	Covariates
**PTSD**			
Ali (2012)	**2.40 (1.13, 5.07)**	living in original house (ref) vs. living in temporary houses	sex, age, family head, employment status, current civil status, income, religious minded, prayer regular, social capital, past-medication history, impairment of work and life, educational status, degree of exposure to earthquake
Asnakew (2019)	**cOR: 2.00 (1.40-2.70)**<br>adjOR: 1.00 (0.70-1.50)	no property destruction (ref) vs. property destructed	sex, age, marital status, history of mental illness, family history of mental illness, experiencing childhood trauma, sustaining physical trauma, witnessing the death of family or friends, witnessing injury of family or friends, thought of death, social support, perceived threat
Cerdá (2013)	1.79 (n.r.)	community sample (ref) vs. residents of IDP camp	age, gender, marital status, number of children, education, mental illness of close one, prior history of trauma, major damage to house, trapped under rubble, injury in earthquake, number of relatives that died, family member with mental illness, loss of job, low social support
Freitag, 2013*	**2.27 (1.60, 3.22)**	"You were driven out of your homeland" no (ref) vs. yes	age, income, gender, family status
Hall, 2019	**2.52 (1.40, 4.54)**	home inhabitable after the typhoon (ref) vs. home uninhabitable	age, gender, region of origin, injury, witness of injury, someone close being injured, almost drowned, saw someone almost drown, saw someone drown, saw dead body, home damaged, personal or family loss of property, 6 types of resource deprivation, 7 types of media use
Khalil, 2024	1.08 (0.58, 2.00)	internally displaced (ref) vs. non-displaced	marital status, living status, age, satisfaction with living status, education level, place of living, gender
Kun et al., 2009	**heavily damaged county: 3.18 (2.07, 4.89)**<br><br>moderately affected county:<br>1.12 (0.69, 1.96)	living in original houses (ref) vs. relocated to shelters or temporary houses	location, gender, age, education, insurance, income, borrowing or loans, death in family, household damage, cultivated land loss
Lopes Cardozo et al., 2000	1.52 (0.68, 3.36)	remained in their homes during the war (ref) vs. internally displaced	rape, forced separation, murder of family/friend, number of trauma events
Scaramutti et al., 2019	**2.94 (1.67, 5.26)**	remained in Puerto Rico (ref) vs. moved to US mainland	age, gender

van Griensven et al., 2006	1.67 (0.72, 3.83)	non-displaced (ref) vs. IDP living in camps	family members, livelihood, suicide, saw ghosts
Wen et al., 2012	**1.69 (1.07, 2.66)**	not displaced (ref) vs. displaced	ethnicity, two-week morbidity, no regular income after earthquake, received mental health support after earthquake, family member died or missing, injured due to the disaster, witnessed someone being killed or injured
**MDD**			
Elhadi, 2022	1.17 (0.99, 1.39)	not displaced due to civil war (ref) vs. displaced within Libya	age, marital status, highest education, working status during COVID-19, COVID-19 infectious status, family member/loved one status, financial issues, domestic violence/abuse, consideration of suicide during lockdown
Freitag, 2013*	1.62 (0.77, 3.41)	"You were driven out of your homeland" no (ref) vs. yes	age, income, gender, family status
Scaramutti et al., 2019	1.67 (0.97, 2.86)	remained in Puerto Rico (ref) vs. moved to US mainland	age, gender
Tiyuri et al., 2023	0.94 (0.55, 1.61)	not living in camp after the flood (ref) vs. temporary living in camps	age, gender, marital status, education, employment status, residence, wealth index, history of mental disorders
**GAD**			
Elhadi, 2022	1.16 (0.91, 1.48)	not displaced due to civil war (ref) vs. displaced within Libya	age, marital status, highest education, working status during COVID-19, COVID-19 infectious status, family member/loved one status, financial issues, domestic violence/abuse, consideration of suicide during lockdown
Scaramutti et al., 2019	1.17 (0.65, 2.11)	remained in Puerto Rico (ref) vs. moved to US mainland	age, gender

Note. CI = 95% confidence interval; *OR* = odds ratio; PTSD = post-traumatic stress disorder. Bold font = statistically significant.
*studies from World War II were included due to the uncertainty of national borders over the course of the war.

Supplement 10: Definition of displacement vs. non-displacement status in comparative studies

Study	Displaced sample	Non-displaced sample
**Ali et al., 2012**	living in temporary houses	living in original house
**Amsalem et al., 2025**	answered “yes” to a question on forced displacement	answered “no” to forced displacement
**Asnakew et al., 2019**	answered “yes” to a question on property destruction	answered “no” to a question on property destruction
**Banal et al., 2010**	families forced to move out of their homes in the Kashmir valley, residing in Muthi camp at Jammu	residents of Muthi Village, Jammu, who had not been displaced and did not live in camp
**Ben-Ezra et al., 2023**	displaced within Ukraine	non displaced residents of Ukraine
**Cerdá et al., 2013**	residents of IDP camp	community sample
**Elhadi et al., 2022**	displaced within Libya	not displaced due to civil war
**Freitag et al., 2011**	displaced during World War II	not displaced during World War II
**Freitag et al., 2013**	answered “yes” to the question "You were driven out of your homeland"	answered “no” to the question "You were driven out of your homeland"
**Greene-Cramer et al., 2020**	displaced due to conflict across Ukraine (excluding Donetsk & Luhansk regions)	Non-displaced adults living in government-controlled conflict-affected regions in eastern Ukraine (Donetsk and Luhansk regions)
**Hall et al., 2019**	home was uninhabitable after the typhoon	home remained inhabitable after the typhoon
**Hamama et al. (2025)**	affected by the Hamas attack on October 7th 2023 and known as internally displaced persons	affected by the Hamas attack on October 7th 2023
**Jayasuriya, 2014**	living in an IDP camp due to the war	never lived in an IDP camp
**Johnson et al., 2022**	displaced either by the Donbass war or the annexation of Crimea	urban-dwelling residents whose status was relatively more stable or secure
**Kakaje et al., 2021**	forced to change area of living due to war (within same city/changing the city/both)	did not change area of living due to war
**Karatzias et al., 2023**	answered “yes” to the question “I had to move to another part of the Ukraine”	answered “no” to the question “I had to move to another part of the Ukraine”
**Kılıç et al., 2006**	relocated to capital	residents of capital who were visitors at time of earthquakes

**Kun et al., 2009**	relocated to shelters or temporary houses	living in original houses
**Kuwert et al., 2009**	answered “yes” to the question “Did you experience forced displacement from the former eastern territories in WWII (e.g. Eastern Prussia, Pomerania, Silesia etc.)?”	answered “no” to the question “Did you experience forced displacement from the former eastern territories in WWII (e.g. Eastern Prussia, Pomerania, Silesia etc.)?”
**Lê et al., 2013**	answered “yes” to the question “Did you move from the place you were living because of Hurricane Katrina?’’	answered “no” to the question “Did you move from the place you were living because of Hurricane Katrina?’’
**Lopes Cardozo et al., 2000**	internally displaced within Kosovo due to the Kosovo war	remained in their homes during the Kosovo war
**Lushchak et al., 2023**	internally displaced within Ukraine due to the war	stayed within their permanent location in Ukraine
**Matsuoka et al., 2023**	relocated to temporary housing or other housing types	did not relocate
**Matthews et al., 2019**	had to leave their home due to the flood	not displaced due to the flood
**Monsalve et al., 2022**	displaced according to the registration with the Central Colombian Register for Victims	not registered as displaced person
**Peleg & Gendelman, 2025**	relocated to safer areas during the Gaza war	not relocated
**Roitblat et al., 2024**	answered “yes” to the question “Did you recently arrive from the east [of Ukraine]?” and “Do you plan to stay in Lviv during the hostilities?”	answered “no” to the question “Did you recently arrive from the east [of Ukraine]?”
**Scaramutti et al., 2019**	displaced Puerto Ricans living in urban and rural/suburban areas in Florida	Puerto Ricans who remained in Puerto Rico
**Shiga et al., 2021**	Fukushima residents who survived the earthquake and faced mandatory evacuation from their residence because of the Fukushima Daiichi Nuclear Power Plant accident	Fukushima residents who survived the earthquake and still live in their own home
**Strauss et al., 2011**	experienced forced relocation and/or displacement from former German eastern territories or other occupied countries before 1946	did not experienced flight and/or displacement
**Takahashi et al., 2016**	lost their homes due to the tsunami and moved to evacuation centres or to family and friend’s houses, most of them later moved to temporary housing	did not you change their house (including a shelter evacuation)
**Tiyuri et al., 2023**	temporarily living in the camps after the flood	not living in camp after the flood
**Tsuchiya et al., 2019**	living in “prefabricated temporary housing”, “private rental housing”, “houses of relatives or acquaintances” or “reconstructed housing”	living in “same housing as that before the GEJE”
**van Griensven et al., 2006**	IDPs living in camps (Phang Nga)	non-displaced persons in the three Thai provinces of Phang Nga, Krabi, and Phuket

**Wani et al., 2020**	answered “yes” to the question "did you have to move out of your home"	answered “no” to the question "did you have to move out of your home"
**Wen et al., 2012**	displaced after earthquake	not displaced after earthquake
**Zasiekina et al., 2023**	displaced at the time of responding	not displaced at the time of responding
